# Virtual reality exposure therapy with graded interviewer reactions for public speaking anxiety in university students: a randomized controlled trial protocol

**DOI:** 10.1186/s13063-026-09779-0

**Published:** 2026-05-19

**Authors:** Myungsung Kim, Min Jeon, Yerin Lee, Yunji Lee, Soeun Yu, Sangil Lee, Hwang Kim, Chul-Hyun Cho, Chung-Yean Cho, Ji-Won Hur, Dooyoung Jung

**Affiliations:** 1 Graduate School of Health Science and Technology, Ulsan National Institute of Science and Technology, Ulsan, Republic of Korea; 2 Department of Biomedical Engineering, Ulsan National Institute of Science and Technology, Ulsan, Republic of Korea; 3 School of Liberal Arts, Ulsan National Institute of Science and Technology, Ulsan, Republic of Korea; 4 Department of Design, Ulsan National Institute of Science and Technology, Ulsan, Republic of Korea; 5 Department of Psychiatry, Korea University College of Medicine, Seoul, Republic of Korea; 6 School of Film and Multimedia, Korea National University of Arts, Seoul, Republic of Korea; 7 School of Psychology, Korea University, Seoul, Republic of Korea; 8 School of Digital Humanities and Computational Social Sciences, Korea Advanced Institute of Science and Technology, Daejeon, Republic of Korea; 9 Mind Care & Growth Center, Korea Advanced Institute of Science and Technology, Daejeon, Republic of Korea

**Keywords:** Public speaking anxiety, Virtual reality exposure therapy, Job interview training, Social presence, Randomized controlled trial

## Abstract

**Background:**

Public speaking anxiety (PSA) significantly impairs academic and professional outcomes among university students. While virtual reality exposure therapy (VRET) shows promise for PSA treatment, the specific contribution of social and emotional interviewer reactions remains unclear. This study examines whether VRET with graded interviewer reactions leads to greater reduction in public speaking anxiety than standard automated VR exposure.

**Methods:**

This two-arm, parallel-group, superiority randomized controlled trial will recruit 92 Korean university students aged ≥18 years with elevated PRPSA (Personal Report of Public Speaking Anxiety-18 ≥ 58). Participants will be randomized 1:1 to receive either VR interview training with graded interviewer reactions or control VR training with automated voice prompts only. The intervention consists of three VR sessions, with assessments conducted at baseline, immediately after each session, and at 6- and 12-week follow-ups. The primary outcome is change from baseline in Public Speaking Anxiety Scale (PSAS) total scores at each post-baseline assessment occasion, analyzed using a linear mixed-effects model including fixed effects for group, assessment occasion, and their interaction. Secondary outcomes include Liebowitz Social Anxiety Scale–Self Report and Fear of Negative Evaluation–Brief scores, as well as physiological indicators reflecting anxiety responses during VR exposure, including heart rate variability and electrodermal activity. Exploratory analyses will examine behavioral synchrony using motion energy analysis of video recordings and acoustic features of speech to capture anxiety-related movement and vocal patterns.

**Discussion:**

This trial addresses the limited evidence regarding the added value of graded interviewer reactions in VRET for public speaking anxiety by examining whether they improve outcomes beyond those achieved with standard automated VR exposure. The findings may clarify the role of socially responsive feedback in virtual exposure and inform the development of VR-based anxiety interventions.

**Trial registration:**

Clinical Research Information Service (CRiS) KCT0011111. Registered on 06 November 2025.

**Supplementary Information:**

The online version contains supplementary material available at 10.1186/s13063-026-09779-0.

## Background

Recognized as one of the most prevalent fears in the general population, Public Speaking Anxiety (PSA) has been shown through extensive research to rival or even surpass the fear of death among Americans [1]. The Diagnostic and Statistical Manual of Mental Disorders, Fifth Edition (DSM-5), includes a performance-only specifier for social anxiety disorder, addressing cases in which anxiety manifests exclusively in evaluative situations [2]. When PSA becomes chronic, the risk of progression to generalized social anxiety disorder increases substantially [3]. In such clinically meaningful cases, PSA extends beyond a simple emotional response to encompass avoidance behaviors, physiological hyperarousal, and cognitive interference [4].

Building on this clinical understanding, past research emphasizes that PSA is particularly pronounced among university students worldwide, representing one of the most common psychological difficulties experienced in academic settings [5, 6]. PSA can have a pronounced and enduring negative impact on students’ academic performance and later professional success [7] and has been identified as a clinically significant concern among healthcare professionals [8]. Moreover, this anxiety often persists beyond the academic environment, manifesting in other social-evaluative contexts that demand self-presentation, such as job interviews. It directly influences students’ transition into the working world, where interview anxiety emerges as a particularly significant barrier in employment processes. Job interviews represent a prototypical PSA situation [9], exemplifying high-stakes contexts in which social performance abilities are directly evaluated by interviewers [10]. Applicants with elevated interview anxiety often display anxious non-verbal behaviors during interviews, which may negatively influence interviewer evaluations and subsequently affect hiring decisions [11, 12]. These effects extend beyond individual career development, adversely impacting organizational talent-selection processes [13].

To counter these detrimental effects of anxiety, practitioners supported by extensive research and clinical evidence have identified exposure therapy as the most effective intervention for interview anxiety [14]. Operating on the principle of reproducing actual performance situations to elicit realistic anxiety responses, this re-enactment of the event gradually reduces anxiety through systematic desensitization achieved via repeated exposure [15].

However, traditional in vivo exposure therapy is constrained by several limitations, including high costs, time requirements, and psychological burden, which may restrict its practical application in clinical decision-making [16]. These barriers have been increasingly addressed through rapidly advancing virtual reality (VR) technology [17]. VR exposure therapy (VRET) represents an innovative treatment approach capable of inducing anxiety levels comparable to real situations, while enabling repeated exposure in safe, controlled environments, thereby effectively overcoming the primary barriers associated with traditional methods. The actual effectiveness of VRET has been empirically supported by recent meta-analytic research. In particular, a meta-analysis conducted by Reeves et al. (2022), which analyzed seven studies comprising 341 participants, demonstrated that VRET produced large effect sizes for PSA reduction compared with control groups (*d* = −1.39, 95% CI = −2.08 to −0.70, *Z* = 3.96, *p* < 0.001) [18]. Importantly, this effect size closely approximated that of traditional in vivo exposure therapy (*d* = −1.41), suggesting that VRET offers comparable clinical utility and may serve as a viable alternative to conventional methods [18].

Regarding VR intervention research targeting interview situations, recent studies have shown promising outcomes. General VR-based presentation training research has reported large effect sizes, stabilization of physiological indicators such as heart rate variability, and improvements in presentation behaviors [19]. Furthermore, research on Virtual Reality Job Interview Training (VR-JIT), specifically designed for employment preparation, has presented not only reductions in interview anxiety but also enhancements in interview skills and increased actual employment rates [20, 21]. These findings reflect high ecological validity, and indicate that VRET can extend beyond simple anxiety reduction to produce tangible functional improvements in job interview performance.

Despite the growing body of VR interview intervention research, existing studies have focused primarily on quantitative variables (such as audience size, presentation venue scale, and distance from the audience) to validate anxiety induction and intervention effects, while omitting social presence, which has been shown to constitute a core element of VRET [22]. Some studies have incorporated social interactions, such as ambiguous or negative audience feedback [23], without determining whether these factors enhance intervention effectiveness [24]. Nevertheless, systematic research on qualitative factors, including interviewers’ emotional and social reactions, remains considerably limited. Graded reaction difficulty adjustments (from positive to neutral to negative) have only been mentioned exploratorily [25], and systematic comparative studies are still lacking.

In addition, exploration of connections between physiological indicators and actual behavioral changes remains insufficient. Recent research reveals significant gaps in understanding how physiological arousal translates to observable behaviors in PSA contexts. Gallego et al. (2022) found that while autonomic indicators are frequently elevated in high-anxiety individuals, “the evidence linking these physiological markers to observable behavioral changes is mixed and often inconsistent,” showing considerable individual variability, where some exhibit high physiological activation without corresponding behavioral changes [4].

Since PSA usually manifests through visually observable behaviors (e.g., gesturing and head movements), objective measurement of these behaviors is necessary. In particular, auditory dimensions, representing an important behavioral component and manifesting through voice tone and vocal tremor, have revealed that anxiety fundamentally reorganizes vocal parameters, with jitter identified as the sole speech parameter consistently linked to the anxiety state. Most voice analysis research has been conducted in controlled laboratory settings with limited ecological validity, focusing on isolated parameters while overlooking the complex interrelationships among vocal features.

In addition, behavior encompasses acoustic and prosodic dimensions, including pitch dynamics, loudness modulation, pause structure, and speech rate [26]. Recent findings have suggested that anxiety reorganizes the multivariate relationships among vocal parameters, rather than producing stable shifts in single acoustic features. Among these parameters, jitter, which quantifies micro-level irregularities in vocal fold vibration, has frequently emerged as a central feature associated with anxious arousal within network-based analyses [27]. However, findings across studies are not entirely consistent, indicating that the role of jitter should be interpreted within the broader interdependence of acoustic features rather than as a standalone marker [27].

Jitter estimation requires highly controlled recording and analysis procedures to ensure measurement reliability, and such methodological sensitivity makes it less feasible in naturalistic speech contexts [28]. Accordingly, the present study has focused on higher-level prosodic features (e.g., pitch variability, loudness modulation, and temporal fluency), which have been repeatedly identified as salient cues of expressive behavior under ecologically valid communicative conditions [26, 28]. Previous voice analysis research has primarily relied on controlled laboratory tasks using acted or scripted speech, often emphasizing isolated acoustic parameters while neglecting higher-order temporal dynamics and inter-feature dependencies [27, 28]. These methodological limitations underscore the need for multimodal frameworks that integrate physiological markers, behavioral synchrony, and prosodic vocal behavior within ecologically valid communicative settings, a direction increasingly emphasized but still underexplored in public speaking anxiety research [28].

Recent studies have furthermore reported that, in social interaction situations, not only individual movements but also bidirectional synchronization with interaction partners correlate with social anxiety states [29]. A deeper understanding is needed of how such synchronization levels relate to anxiety induction and intervention effects in interview situations. This area has not yet been systematically investigated in PSA intervention research. Therefore, these limitations highlight the need for multimodal approaches that examine physiological markers, behavioral synchronization, and vocal characteristics within ecologically valid VR contexts.

This study employs a randomized controlled trial (RCT) to investigate how the presence or absence of interviewers’ social and emotional reactions in interview situations affects participants’ short-term anxiety induction and long-term intervention effects. The primary objective is to test whether VRET with graded interviewer reactions produces greater reductions in public speaking anxiety than standard automated VRET. Specifically, through multidimensional analyses of PSA-related self-report scales and physiological and behavioral indicators between groups, we seek to comprehensively examine how interviewer reactions in interview situations influence intervention effectiveness. We hypothesize that participants assigned to the graded-reaction intervention will show greater improvement in public speaking anxiety across prespecified assessment occasions than those assigned to the control condition.

## Methods

### Trial design

This study is a two-arm, parallel-group, superiority randomized controlled trial with 1:1 allocation, comparing PSA improvement between VR presentation simulations incorporating interviewers’ social and emotional reactions and VR presentation simulations without interviewer reactions [30]. Repeated assessments will be conducted over 12 weeks, including baseline, post-session assessments after each of the three intervention sessions, and 6- and 12-week follow-ups.

### Participants

This study will be conducted with students from a single university in Ulsan who report PSA. Participants will be recruited through promotional posters within the university and online community promotional posts. Students wishing to participate will first complete online self-screening to assess preliminary inclusion and exclusion criteria. Participants must be university students aged 18 years or older, native Korean speakers, with Personal Report of Public Speaking Anxiety-18 (PRPSA-18) scores of 58 points or higher [31]. The PRPSA-18 is an 18-item empirically developed scale designed to screen individuals with elevated public speaking anxiety levels. The 58-point cutoff represents the optimal criterion for distinguishing high PSA [31]. Exclusion criteria include individuals currently receiving treatment for PSA or social anxiety disorder, those receiving psychiatric diagnosis and medication treatment, individuals diagnosed with major depressive disorder, panic disorder, or agoraphobia, those at risk for suicidal ideation, and individuals with VR usage limitations (visual impairment, severe dizziness, etc.). Those who are eligible will be invited to the laboratory, where a study investigator will explain the study, confirm eligibility, and obtain written informed consent. After enrollment, participants will be randomized in a 1:1 ratio to one of the two study arms. Baseline assessment (T0) will then be conducted before the first intervention session, after which participants will proceed to the intervention and subsequent follow-up assessments. Expected demographic characteristics of the study population are summarized in Table 1.

**Table 1:** Summary table of demographic participant characteristics for a protocol

Non-clinical participant characteristics	
Characteristics	The people we would expect to see included
Age	Mean age 20–22 years (range 18–25 years). The average age of Korean university students is 18–19 years for freshmen, with most enrolled students expected to be distributed in their early twenties [32]
Sex	Given the predominantly male composition of the science and engineering student population (73.3% male, 26.7% female), and evidence that public speaking anxiety is typically higher among females [33], the gender distribution of the study sample is expected to be more balanced
Gender	Most participants are expected to have gender identity consistent with sex assigned at birth
Race, ethnicity and ancestry	Limited to native Korean speakers according to inclusion criteria to ensure cultural and linguistic homogeneity
Socioeconomic status	Expected to be predominantly middle class. The socioeconomic background of university students in the Ulsan region is at a moderate level, with most predicted to fall within the household income range of 4–6 million KRW per month [33]
Geographic location	Predominantly residents of Ulsan Metropolitan City and nearby Gyeongsangnam-do region

### Sample size

The primary outcome is public speaking anxiety as measured by the PSAS, assessed at six prespecified assessment occasions (baseline; immediately after each of the 3 sessions; 6- and 12-week follow-ups). The study is powered to detect a group-by-assessment-occasion interaction reflecting between-group differences in change from baseline in PSAS across repeated assessments.

Previous meta-analyses demonstrate that VRET produces large effects versus waitlist conditions (*g* = 0.90) and shows comparable efficacy to in vivo exposure therapy [16, 34]. However, since both groups in the present trial receive active PSA training, we anticipated only a small incremental effect. Our intervention extends standard VR exposure by introducing graded interviewer reactions delivered as sequential components of a single exposure package. Research indicates that social-evaluative characteristics of virtual audiences can influence performance confidence and anxiety responses, with some improvements transferring to real-world public speaking performance [25, 34].

**Fig. 1:**
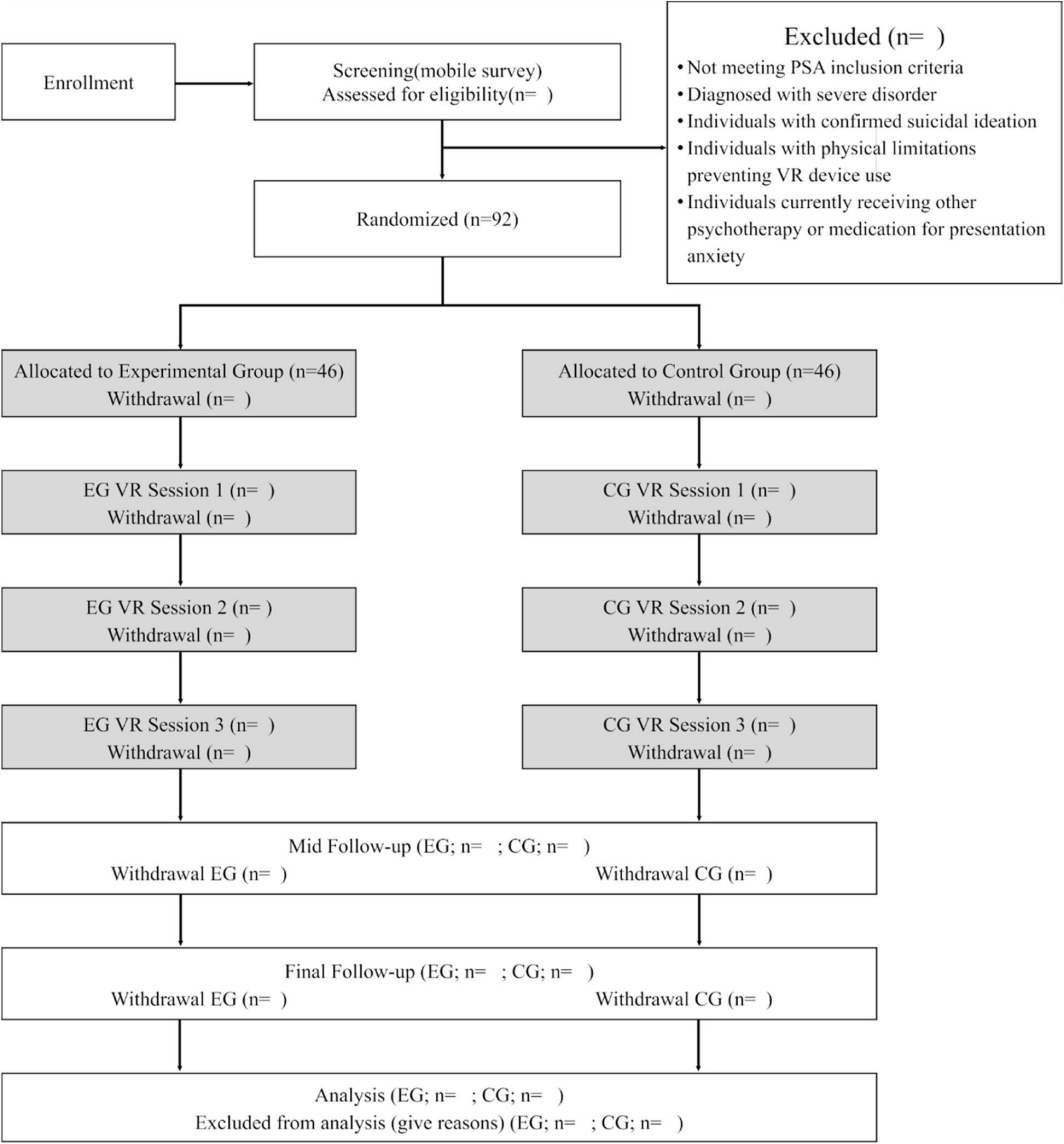
The CONSORT flow diagram. EG, experimental group; CG, control group; white boxes indicate procedures common to both groups; grey boxes indicate group-specific procedures

Based on these findings, a conservative effect size of *f* = 0.15 was assumed for between-group differences in repeated outcome change. A priori power analysis using G*Power (ANOVA: repeated-measures, within-between interaction; *α* = 0.05, 1 − β = 0.80, two groups, six measurements, correlation among repeated measures = 0.50, nonsphericity correction *ε* = 0.50) indicated a total sample of *N* = 78. Although the primary analysis will use a linear mixed-effects model, the sample size was calculated using a repeated-measures ANOVA framework focusing on the group-by-assessment-occasion interaction. This interaction corresponds to the primary estimand of interest and aligns with the primary analysis model. The ANOVA-based approach was adopted as an approximation because prior data were insufficient to parameterize variance components required for LMM-based simulation. To account for approximately 15% attrition commonly observed in VR exposure studies [35], the recruitment target was set at 92 participants (46 per group). Figure 1 shows the CONSORT flow diagram for the recruitment process.

### Procedure

#### Screening

Prospective participants initially complete the PRPSA-18 through a custom-developed webpage to assess their PSA levels and undergo additional online self-screening. Participants meeting inclusion criteria with scores of 58 points or higher receive detailed study information and are invited to the laboratory for in-person eligibility confirmation. During screening, participants are also assessed for exclusion criteria including current diagnosis of severe mental illness, ongoing psychiatric treatment, or presence of suicidal ideation (defined as scoring ≥ 1 on item 9 of the PHQ-9). Individuals meeting any exclusion criteria are not eligible for study participation.

At the laboratory visit, a study investigator confirms eligibility, explains the study, and obtains written informed consent. Participants are then enrolled and randomized to the experimental or control group in a 1:1 ratio using computer-generated allocation. Baseline assessment (T0) is conducted after randomization and before the first intervention session. To minimize noise potentially affecting physiological indicator measurements, participants are advised to maintain adequate sleep and limit caffeine and alcohol consumption during the 1-week period from study enrollment through intervention completion. This lifestyle management serves to enhance measurement accuracy of physiological indicators related to autonomic nervous system function.

#### Pre-session voice recording and stabilization before each session

Participants receive brief study explanations before donning Head-Mounted Displays (HMD) and physiological monitoring equipment (Shimmer3 GSR + unit; Shimmer Sensing, Dublin, Ireland). Following equipment setup, participants produce 1) a 5-s sustained vowel/a/(“ah ~ ”; the central 3 s analyzed) and 2) a continuous reading passage from the standardized Korean paragraph “Fall” (Kim, 1996; see Appendix A for full text, IPA transcription, and English translation), which provides balanced phonemic coverage and is widely used for voice and speech assessments [36]. These recordings will be analyzed for fundamental frequency (F0), intensity, pause duration, and speech rate as baseline prosodic indicators. The sustained vowel duration aligns with CAPE-V recommendations for stable phonation [37]. The passage length was selected to ensure sufficient voiced material for reliable acoustic and temporal analyses, as long-term speaking-rate measures typically stabilize within ~8–16 s [38].

After the voice recording, participants undergo a 3-min stabilization period during which they fixate on a small black dot displayed at the center of a blank VR screen. This stabilization phase allows physiological indicators to reach baseline levels and facilitates adaptation to the VR environment. The interview simulation then proceeds automatically. At Session 1, these measures form part of the study baseline assessment (T0); analogous pre-session recordings in Sessions 2 and 3 are used only for within-session standardization and are not additional baseline assessments.

#### First session

**Fig. 2:**
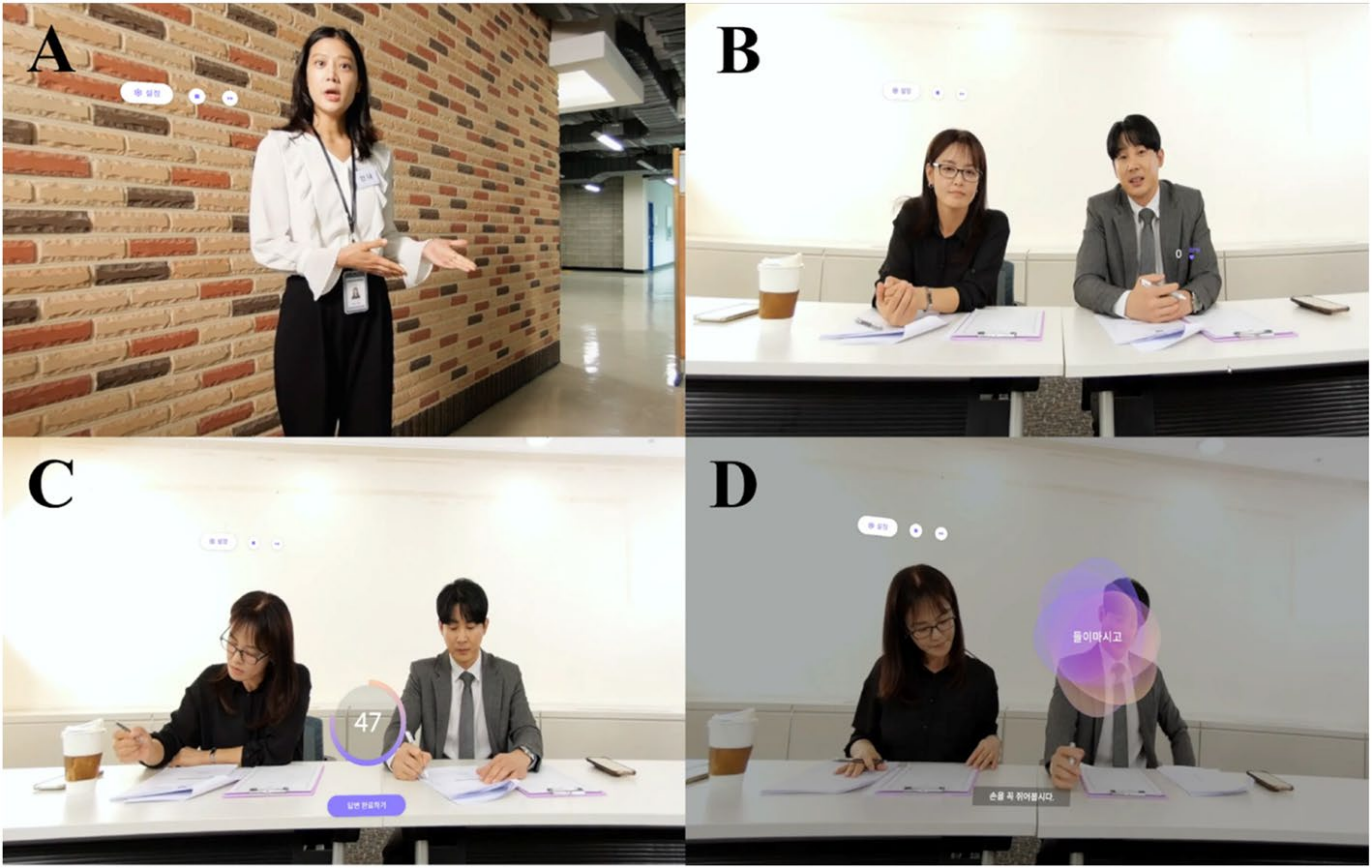
Actual VR screen captures as viewed by participants. **A** Situation where participants wait and receive entry guidance. **B** Face-to-face situation with the interviewer. **C** Timer interface displayed when responding to interview questions. **D** Relaxation training interface immediately following interview simulation

When the VR content begins, participants first wait in a virtual waiting area where they receive entry guidance through on-screen and audio instructions. After this brief orientation, the interviewer requests a brief self-introduction. Upon receiving the question, participants are given a 20-s preparation period (indicated by an on-screen countdown timer) to organize their thoughts before beginning their response. Participants then present for 1 min while monitoring a timer interface. In the experimental group (EG) intervention, the interviewer demonstrates positive reactions such as nodding while listening to participants’ self-introductions and responds with “Yes, I heard you well.”, “Please relax and feel free to speak comfortably.” upon presentation completion. In the control group (CG), interview questions are provided through automated voice prompts, with no visual or auditory interviewer reactions provided. Both groups participate in a 2-min “positive self-talk session” immediately following presentation simulation, after which equipment is removed and questionnaire surveys are completed. Participants are scheduled to revisit the laboratory for the second session after 48 h (± 2 h). Figure 2 shows screen captures of what participants observe through VR.

#### Second session

Participants undergo the same stabilization session as the first session before proceeding with the interview. In the experimental group, the interviewer requests an explanation of strengths and weaknesses with a neutral expression. Participants receive a 20-s preparation period followed by 1 min for their presentation. The interviewer maintains neutral observation of the participant before examining application materials and responding “Hmm… I see” upon presentation completion. In the control group, interview questions are provided through automated voice prompts with no visual or auditory interviewer reactions. Both groups participate in a 2-min “worry reflection and acceptance practice” immediately following presentation simulation.

#### Third session

**Fig. 3:**
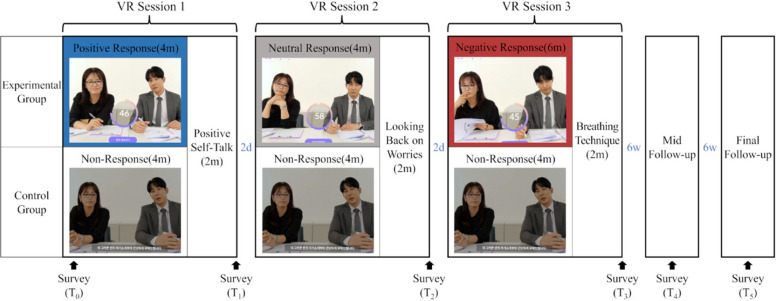
Schematic representation of session-specific intervention components and questionnaire administration timepoints across three VR sessions. The experimental group receives graded interviewer reactions progressing from positive (Session 1) to neutral (Session 2) to negative responses (Session 3), while the control group receives automated voice prompts without visual or social-emotional interviewer reactions. Across groups, the interview structure, topics, preparation periods, and speaking durations are identical; only the interviewer reaction format differs. Each session includes specific coping strategies: positive self-talk (Session 1), worry reflection and acceptance (Session 2), and breathing/muscle relaxation techniques (Session 3). Surveys are administered at baseline (T₀) and immediately after each session (T₁, T₂, T₃), with follow-ups at 6 weeks (T₄) and 12 weeks (T₅)

Participants proceed with interviews following the stabilization session. In the experimental group, the interviewer displays a tired expression, sighs, makes notes on paper, and asks “Next, please tell me about the two most memorable news items you’ve seen recently and explain your reasons.” After a 20-s preparation period, participants present for 1 min. During the participant’s response, the interviewer begins whispering to another interviewer, then responds with negative expression and voice tone upon response completion: “I can’t hear your voice well, so please speak a little louder and more clearly. Also, could you explain what you mentioned in more detail?” In the control group, interview questions are provided through automated voice prompts with no visual or auditory interviewer reactions. Both groups participate in a 2-min “breathing and muscle relaxation training” immediately following presentation simulation. Across groups, the interview topics, preparation time, speaking duration, and session sequence are identical; only the interviewer reaction format differs between conditions. The positive, neutral, and negative interviewer reactions were designed as sequential components of a single graded exposure intervention rather than as separate intervention arms. Figure 3 shows the intervention process and questionnaire administration timepoints.

#### Follow-up with open-ended questions

**Fig. 4:**
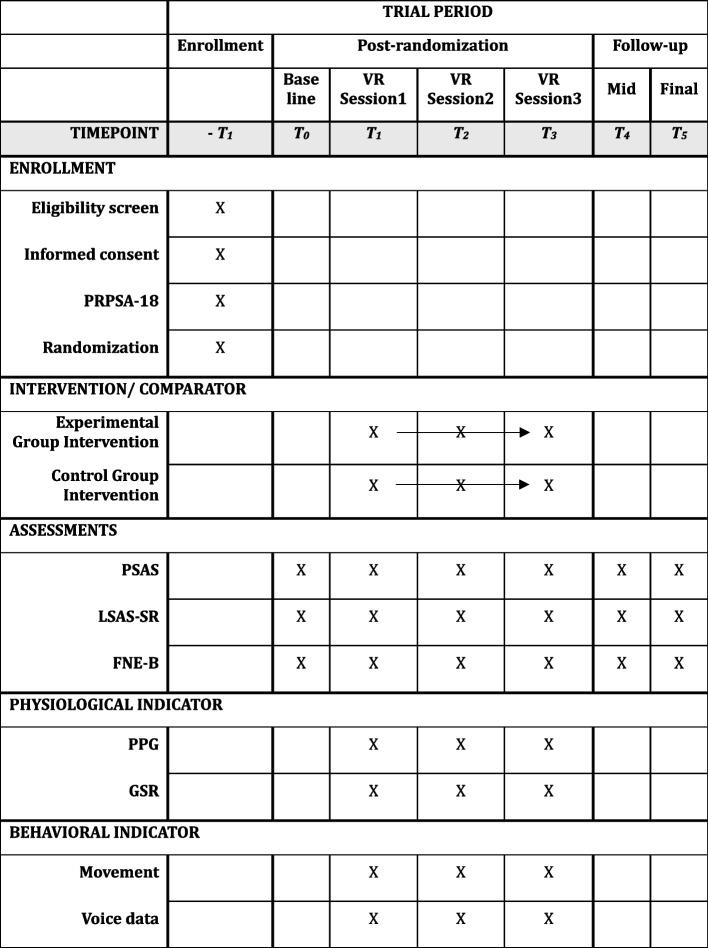
SPIRIT schedule of enrollment, interventions, and assessments throughout the study timeline. Timepoints are designated as follows: -T1 (online self-screening, in-person eligibility confirmation at the laboratory, written informed consent and randomization), T0 (baseline assessment), T1–T3 (immediate post-session assessments during the three VR intervention sessions), T4 (6-week follow-up), and T5 (12-week follow-up). The intervention phase (T1–T3) includes both experimental group (graded interviewer reactions) and control group (automated prompts) protocols. Primary outcomes (PSAS, LSAS-SR, FNE-B) are measured at all six timepoints, while physiological indicators (PPG, GSR) and behavioral measures (movement, voice data) are collected only during the intervention sessions (T1–T3). X marks indicate when each assessment or procedure is conducted

Both groups complete questionnaires remotely at 6 and 12 weeks following the third intervention. Questionnaires include open-ended items allowing participants to freely provide feedback on whether they experienced actual presentation situations such as classes or interviews after the intervention and, if so, in what aspects they perceived intervention effects. Figure 4 shows intervention timepoints and data collection timepoints throughout the experimental process.

#### Participant timeline: schedule of enrollment, interventions, and assessments

### Outcome measurement

#### Primary outcome

Public Speaking Anxiety Scale (PSAS): The primary outcome measure is change from baseline in Korean-version PSAS total scores at each post-baseline assessment occasion (T1–T5), with assessments conducted at baseline (T0), immediately after each intervention session (T1, T2, and T3), and at 6-week (T4) and 12-week (T5) follow-ups. The PSAS is a 17-item self-report questionnaire comprising cognitive, behavioral, and physiological dimensions of public speaking anxiety [39]. The scale demonstrates good psychometric properties and is well-suited for monitoring public speaking anxiety treatment effects [40]. The primary estimand is the between-group difference in mean change from baseline in PSAS total scores across post-baseline assessment occasions, estimated using a linear mixed-effects model with fixed effects for group, assessment occasion, and their interaction.

#### Secondary outcomes

Liebowitz Social Anxiety Scale-Self Report (LSAS-SR): Used to measure social anxiety which is highly related to public speaking anxiety. LSAS-SR is a 48-item scale assessing anxiety and avoidance levels across 24 social situations, with the Korean version validated for university students [41]. The scale demonstrates excellent internal consistency (*α* = 0.92–0.95) [41]. LSAS-SR total scores will be summarized at the group level and compared across the same six assessment occasions used for the primary outcome (T0–T5). 

Fear of Negative Evaluation-Brief (FNE-B): A 12-item scale measuring fear of negative evaluation. Originally developed by Watson and Friend (1969) and shortened by Leary (1983) [42], the Korean version was validated by Lee and Choi (1997) [43]. Items are rated on a 5-point Likert scale, with higher scores indicating greater fear of negative evaluation [44]. FNE-B total scores will be summarized at the group level and compared across the same six assessment occasions used for the primary outcome (T0–T5). 

Physiological indicators: To objectively compare short-term anxiety induction levels and long-term desensitization patterns between groups during exposure, photoplethysmography (PPG) for heart rate measurement and galvanic skin response (GSR) measurement are implemented [45]. For cardiac autonomic activity, the principal variables will include RMSSD and SDNN (log-transformed if distributional assumptions are violated), and pNN50, derived from PPG signals collected from the earlobe at 256 Hz. For electrodermal activity, the principal variables will include mean tonic skin conductance level (SCL) and phasic SCR peak amplitude and peak count derived from GSR signals collected from the index and middle fingers of the non-dominant hand at 256 Hz. These participant-level physiological variables will be summarized by session and compared between groups across intervention stages. Signals will be processed using third-order Savitzky-Golay filters to preserve signal strength while providing smoothing [46]. Heart rate variability reflects autonomic nervous system responses to stress and anxiety [47], while galvanic skin response serves as an indicator of sympathetic nervous system arousal levels [48].

#### Exploratory variables

Behavioral indicators: Intervention effects on behaviors such as movement and vocal changes are examined in exploratory analyses. Movement is collected through fixed cameras filming participants’ upper body frontal views, with Motion Energy Analysis (MEA) used to analyze synchronization patterns with VR interviewer movements [49]. Synchronization patterns are modeled as sine wave dynamics, with amplitude and vertical offset parameters extracted as indicators of interpersonal coordination quality [49]. These synchrony metrics are correlated with session-specific anxiety levels (derived from concurrent physiological and self-report measures) and compared across sessions and participants to examine relationships between social synchrony and anxiety reduction.

Voice data from presentation speech are collected through VR microphones during sessions. Prosodic voice features such as fundamental frequency (f0), intensity contours, pause time, and speech rate will be extracted from recordings during pre-session stabilization. Speech samples will be preprocessed with noise filtering but not segmented into fine-grained phonetic units. Recordings are captured using the Meta Quest 3 headset’s built-in microphone (mono, 48 kHz). Acoustic analyses will be conducted in Praat (v6.3 or later), and all acoustic features will be standardized within participants relative to the pre-session baseline to control for inter-individual variability.

#### Ethics statement

The research protocol was approved by the UNIST Institutional Review Board (UNISTIRB-25–049-A) on October 31, 2025. This study was designed according to the Declaration of Helsinki, Good Clinical Practice guidelines, and SPIRIT reporting guidelines [50]. Study interventions are considered low-intensity, low-risk interventions totaling approximately 1 h over 1 week. Participation is entirely voluntary and participants may discontinue the experiment at any time. Data are pseudonymized with participant identification numbers. Participants receive 40,000 KRW (approximately $28 USD) compensation for study participation.

Expected adverse events include cybersickness-related symptoms (e.g., nausea, dizziness, headache, and visual discomfort) and transient increases in anxiety during VR exposure. Unexpected adverse events will also be documented. Harms will be identified through both spontaneous participant report and structured post-session inquiry. All identified adverse events will be reviewed by the clinical psychologist and psychiatrist investigators and classified according to severity, expectedness, and relatedness to the intervention. Participants who discontinue the intervention because of adverse effects will, whenever feasible, still be invited to complete follow-up assessments.

#### Randomization and blinding

After in-person eligibility confirmation and written informed consent at the laboratory visit, participants are randomly allocated to experimental or control groups in a 1:1 ratio. Computer-generated randomization using Python employs permuted block randomization to allocate 46 participants to each group. Randomization occurs after enrollment and before baseline assessment (T0) and first intervention session. Participants remain blinded to group allocation, understanding they are participating in VR-based interview presentation and relaxation training research without knowing whether they belong to experimental or control groups. While researchers are not blinded during experimental procedures, data analysis and report writing are completed with participant group information blinded, with interpretation beginning only thereafter. Future outcome reporting papers will present blinded report versions as reference materials.

#### Confidentiality and data management

Study data will be stored on encrypted, password-protected laboratory computers with restricted access. Personal identifiers and research data will be stored separately using coded study identification numbers. The file linking participant identities to study IDs will be maintained separately and will be accessible only to the designated monitoring investigator. Data quality will be monitored through ongoing review of study records and weekly cross-checks of questionnaire, physiological, and scheduling data. Secure monthly backups will be performed. Access to the final de-identified dataset will be limited to the first author and the monitoring investigator unless additional access is required for approved analyses.

#### Data preparation and planned analyses

Missing participant data are included in analyses for intention-to-treat (ITT) analysis. ITT analysis preserves randomization principles while reflecting actual clinical environments, analyzing all randomly allocated participants according to their original group assignments regardless of dropout or protocol violations [51]. Primary and secondary analyses of self-report questionnaires outcomes will be conducted using linear mixed-effects models, with fixed effects for group, assessment occasion, and their interaction, and participant-specific random effects to account for within-subject correlation across repeated measurements.

PPG data undergo preprocessing using third-order Savitzky-Golay filters, with heart rate variability analyzed in the time domain to capture short-term autonomic responses. Primary analyses compare PPG-derived, time-domain HRV and heart rate measures between groups during key intervention phases (interviewer questioning, presentation, and stabilization) to assess short-term autonomic reactivity. The primary outcome is ΔRMSSD (presentation minus baseline stabilization), representing parasympathetic reactivity. Secondary analyses include comparisons of mean HR, ΔHR, SDNN, and pNN50 to capture overall autonomic activation and variability. Long-term habituation patterns will be examined by modeling within-subject slopes of ΔRMSSD and ΔHR across the three sessions, with an anticipated progressive reduction in reactivity in the experimental group.

GSR signals are decomposed into tonic (SCL) and phasic (SCR) components using Continuous Decomposition Analysis [48, 52]. For between-group comparisons, phasic response magnitude to interviewer questions are analyzed across sessions. Tonic arousal levels are compared between groups throughout the entire intervention period. Within the experimental group, GSR responses across the sequential positive, neutral, and negative interviewer-reaction stages will be explored in relation to concurrent self-report and behavioral anxiety indicators. Because these stages are delivered in a fixed sequence as part of a single graded intervention package, these analyses will be interpreted as exploratory and will not be used to infer the independent causal effect of each reaction valence.

Motion Energy Analysis (MEA) quantifies movement patterns using 10 frames per second video conversion and gray-scale pixel differencing methodology [49]. Motion energy vectors undergo standard smoothing and z-transformation procedures. Given that control group interviewers remain static, synchronization analysis focuses on within-experimental group patterns. Following established MEA protocols, 4-min interaction segments (question → presentation → interviewer reaction) are analyzed using 30-s sliding windows with cross-correlations computed for time-lags up to ±5 s in 0.1-s steps [49].

Voice analysis examines 1-min presentation recordings using automated prosodic feature extraction. Speech samples will be preprocessed with noise filtering but not segmented into fine-grained phonetic units, as analyses focus on suprasegmental prosodic patterns. Primary measures include fundamental frequency (F0) mean, speech rate, and pause ratio, which together reflect expressive and arousal-related vocal behavior. All features will be within-subject standardized relative to baseline readings to control for inter-individual variability. Voice features will be compared between groups and across sessions, with correlation analyses examining relationships between prosodic stress indicators (e.g., elevated F0, reduced speech rate, increased pause ratio) and concurrent anxiety measures. Expected patterns include higher F0 and slower, less fluent prosodic dynamics in early sessions, followed by progressive normalization indicating reduced performance anxiety [53].

## Discussion

Public speaking anxiety is one of the most common psychological difficulties among university students; however, its prevalence often leads to underestimation of its detrimental impact. Interview situations particularly represent existential problems that students most worry about while also having to actually confront in the near future. While existing VRET has demonstrated substantial effect sizes compared to control groups [8], systematic exploration of additional therapeutic effects from social and emotional audience reactions identified as core elements has been limited.

This study proposes investigating whether PSA induction and desensitization can be enhanced by gradually modulating interviewer emotional reactions in existing VRET simulating interview situations. By comparing this with digital active control groups rather than no-treatment control groups, we seek to ensure experimental ethical validity while clearly deriving additional effects of audience reactions [54]. Secondary and exploratory physiological and behavioral indicators are designed to quantitatively and objectively track anxiety induction levels and patterns during intervention processes beyond pre-post intervention effect differences between groups.

If effectiveness is demonstrated, this study may provide evidence for the importance of social and emotional audience reactions in VRET but also bring various benefits to future intervention development and validation. First, this trial targets PSA as a prevalent and highly relevant symptom before testing VRET with clinical patients (e.g., those diagnosed with social anxiety disorder), serving as evidence for clinical population interventions [55]. Such intervention research in subclinical populations is known to serve as effective bridges to clinical populations [56].

Second, because audience emotional reactions differ from quantitative elements such as audience size and room scale primarily addressed in existing VRET effectiveness validation studies [21], they present new research directions enabling study expansion through various reaction scenarios. Social presence constitutes a core element of VRET, reportedly capable of producing greater therapeutic effects when emotional reactions are included [57]. This study’s results will contribute to future research design and interpretation by providing physiological and behavioral indicators alongside PSA improvement and how anxiety is induced and desensitized.

This study has several limitations. First, interviewer reactions in the experimental condition are delivered in a fixed positive-to-neutral-to-negative sequence. Accordingly, the present design evaluates the overall graded exposure package rather than the isolated effect of each reaction valence. Although this sequencing may enhance ecological validity, it precludes independent estimation of the contribution of each component.

Second, intervention frequency and exposure duration may be insufficient. This reflects our purpose of confirming PSA improvement and maintenance levels through brief interventions rather than excessively lengthy interventions, given that the target population consists of general university students reporting only PSA [19]. However, prior research findings support that brief interventions can also be effective for PSA [58]. Future application to clinical populations will require preliminary experiments adjusting frequency and exposure duration with clinical groups.

Third, self-report questionnaires used as primary outcomes in this study are retrospective and contain potential for individual bias [59]. For example, despite experiencing high anxiety during exposure, participants may provide false reports due to social desirability bias or self-protective attitudes after exposure completion [60]. To address these limitations, physiological indicators are simultaneously employed, which not only track objective anxiety levels during interventions but also provide participants with awareness that objective measurement is occurring, inducing more honest evaluations [61].

Fourth, several methodological considerations should be acknowledged. The recruitment of Korean-speaking students from a single engineering-focused college may limit generalizability across cultural contexts and educational settings, with potential gender imbalance in the sample. While VR provides controlled experimental environments, inherent limitations in replicating full social presence and the artificiality of HMD-mediated interactions may affect ecological validity relative to real-world interview situations. Additionally, physiological indicators are subject to individual variability and environmental factors that may not be fully controlled, and the 12-week follow-up duration, while adequate for assessing medium-term effects, may not capture longer-term intervention maintenance.

Overall, this study’s results can provide important information regarding how to design and measure effects of social and emotional audience reactions as core factors in VRET for future research and clinical practice. This may contribute to intervention development suited to more diverse situations and individual characteristics as new, measurable components of VRET [62]. Furthermore, physiological and behavioral indicators derived from this study may serve as foundational data for developing personalized VRET.

Beyond verifying these additional effects, this study aims to clarify how social and emotional feedback contributes to desensitization as a therapeutic mechanism within virtual environments. If a significant group × assessment-occasion interaction is observed, it would suggest that adding graded social feedback to standard VR exposure may contribute to greater anxiety reduction over the course of the intervention and follow-up period. However, because interviewer reactions are delivered in a fixed sequence, such findings should be interpreted as evidence for the overall graded package rather than for the isolated effect of any single reaction valence.

From a clinical perspective, although adaptive VRET was not the primary focus of this study, the findings may extend existing personalization approaches by providing empirical support for future adaptive VRET systems that adjust the interviewer’s emotional expressions and feedback intensity according to participants’ physiological reactivity or behavioral synchrony patterns. This adaptive approach could enhance ecological validity and engagement, while supporting scalable and cost-effective digital interventions applicable to a wide range of individuals, including those with social anxiety disorder. In a broader context, the study may help redefine VRET as a socially interactive rather than purely exposure-based form of therapy.

### Trial status

This clinical trial was registered with the UNIST Institutional Review Board on October 31, 2025 (UNISTIRB-25–049-A). Subsequently, it was registered in the Clinical Research Information Service on November 6, 2025 (KCT0011111), for international journal submission. Recruitment procedures will commence on November 20, 2025, and conclude on December 31, 2025. This is the first protocol publication for this trial, and no separate Korean-language protocol paper has been published. Any deviations from the study protocol will be fully documented, and the protocol will be updated in the trial registry accordingly.

### Dissemination and authorship policy

The results of this trial will be disseminated through peer-reviewed publications and academic conference presentations. Authorship for the main trial report and any future secondary or exploratory publications will be determined according to the International Committee of Medical Journal Editors (ICMJE) authorship criteria and will be based on substantive intellectual contribution. No professional medical writing support will be used. If external English-language editing is obtained, it will be acknowledged appropriately and will not confer authorship.

## Supplementary Information

Supplementary Material 1.

## Data Availability

The datasets generated and analyzed during this study will be made available from the corresponding author on reasonable request following completion of the trial and primary publication of results.

## Abbreviations

ANOVA: Analysis of variance
CG: Control group
DSM-5: Diagnostic and Statistical Manual of Mental Disorders, Fifth Edition
EG: Experimental group
F0: Fundamental frequency
FNE-B: Fear of Negative Evaluation-Brief
GSR: Galvanic skin response
HMD: Head-mounted display
ITT: Intention-to-treat
LSAS-SR: Liebowitz Social Anxiety Scale-Self Report
MEA: Motion energy analysis
PPG: Photoplethysmography
PRPSA-18: Personal Report of Public Speaking Anxiety-18
PSA: Public speaking anxiety
RCT: Randomized controlled trial
VRET: Virtual reality exposure therapy
VR: Virtual reality
VR-JIT: Virtual Reality Job Interview Training
