# Supplementary material for: Virtual reality exposure therapy with graded interviewer reactions for public speaking anxiety in university students: a randomized controlled trial protocol
Source: Trials. 2026 May 19;27:485. doi: 10.1186/s13063-026-09779-0 (PMC13352607; doi:10.1186/s13063-026-09779-0)
Supplement: Supplementary file 1 — Supplementary Material 1. [file 13063_2026_9779_MOESM1_ESM.zip › Appendix A_251110.docx]

**Appendix A. Standardized Korean reading passage for voice and speech assessment**

Origianl language(Korean) with International Phonetic Alphabet

| 우리나라의 가을은 참으로 아름답다. /u.ɾi.na.ɾa.ɰi ka.ɯ.ɾɯ.nɯn tɕʰa.mɯ.ɾo a.ɾɯm.tap.t͈a/  무엇보다도 산에 오를 땐 더욱 더 그 빼어난 아름다움이 느껴진다. /mu.ʌt̚.p͈o.da.do sa.ne o.ɾɯl t͈ɛn tʌ.uk t͈ʌ kɯ p͈ɛ.ʌ.nan a.ɾɯm.da.u.mi nɯ.k͈jʌ.t͡ɕin.da/  쓰다듬어진 듯한 완만함과 깎아 놓은 듯한 뾰족함이 어우러진 산등성이를 따라 오르다 보면 /s͈ɯ.da.dɯ.mʌ.t͡ɕin tɯ.tʰan wan.man.ham.ɡʷa k͈a.k͈a no.ɯ.nɯn tɯ.tʰan p͈jo.t͡ɕo.kʰa.mi ʌ.u.ɾʌ.t͡ɕin san.dɯŋ.sʌŋ.i.ɾɯl t͈a.ɾa o.ɾɯ.da po.mjʌn/  절로 감탄을 금할 수가 없게 된다. /t͡ɕʌl.ɾo kam.tʰa.nɯl kɯm.hal su.ɡa ʌp̚.k͈e dwen.da/  붉은색, 푸른색, 노란색 등의 여러 가지 색깔이 어우러져 타는 듯한 감동을 주며 /pul.ɡɯn.sɛk̚ pʰu.ɾɯn.sɛk̚ no.ɾan.sɛk̚ tɯŋ.ɯi jʌ.ɾʌ ka.t͡ɕi sɛk̚.k͈a.ɾi ʌ.u.ɾʌ.t͡ɕjʌ tʰa.nɯn tɯ.tʰan kam.doŋ.ɯl t͡ɕu.mjʌ/  나아가 신비롭기까지 하다. /na.a.ɡa ɕin.bi.ɾop̚.k͈i.k͈a.t͡ɕi ha.da/  숲 속에 누워서 하늘을 바라보라. /sup̚ so.ɡe nu.wʌ.sʌ ha.nɯ.ɾɯl pa.ɾa.bo.ɾa/  쌍쌍이 짝지어 있는 듯한 흰 구름, 높고 파란 하늘을 쳐다보고 있노라면 /s͈aŋ.s͈aŋ.i t͡ɕ͈ak̚.t͡ɕi.ʌ it̚.nɯn tɯ.tʰan hɰin ku.ɾɯm nop̚.k͈o pʰa.ɾan ha.nɯ.ɾɯl tɕʰʌ.da.bo.ɡo it̚.no.ɾa.mjʌn/  과연 옛부터 가을을 천고마비의 계절이라 일컫는 이유를 알게 될 것만 같다. /kʷa.jʌn jet̚.pʰu.tʰʌ ka.ɯ.ɾɯl tɕʰʌn.ɡo.ma.bi.ɯi kje.t͡ɕʌ.ɾi.ɾa il.kʰʌ.nɯn i.ju.ɾɯl al.ɡe dweɭ kʌt̚.man kat̚.t͈a/ |
| --- |

English Translation and Semantic Content

| Autumn in our country is truly beautiful. Above all, its outstanding beauty is felt even more when climbing mountains. As you climb along the ridges where gentle slopes caressed by nature blend with sharply carved peaks, you cannot help but admire spontaneously. Various colors—red, green, yellow—blend together, giving a burning impression and even appearing mystical. Lie down in the forest and look at the sky. Gazing at the white clouds seeming to pair up and the high, blue sky, you will come to understand why autumn has been called the season of 'high sky and fat horses' since ancient times. |
| --- |
