# Supplementary material for: Virtual reality exposure therapy with graded interviewer reactions for public speaking anxiety in university students: a randomized controlled trial protocol
Source: Trials. 2026 May 19;27:485. doi: 10.1186/s13063-026-09779-0 (PMC13352607; doi:10.1186/s13063-026-09779-0)
Supplement: Supplementary file 1 — Supplementary Material 1. [file 13063_2026_9779_MOESM1_ESM.zip › Supplementary2_IRB Approval_Initial (Korean original with official seal).pdf]

심의결과통지서

**과제번호**	2025-08-005	**IRB 승인번호**	UNISTIRB-25-049-A
**연구제목**	(국문) 사회평가적 위험 상황에서의 가상현실 면접 노출치료가 발표불안에 미치는 효과: 상황적 요소와 상호작용 요소를 고려한 무작위 대조 시험<br>(영문) Effects of Virtual Reality Interview Exposure Therapy on Public Speaking Anxiety in Social-Evaluative Threat Situations: A Randomized Controlled Trial Considering Situational and Interactive Factors		
**연구책임자**	**성명**: 정두영	**직위**	교수
	**소속**: UNIST/ 헬스케어센터		
**심의종류**	■ 초기심의 □ 재심의(보완, 이의신청) □ 중간보고 □ 변경 □ 기타:		
**연구유형**	■ 인간대상연구 □ 인체유래물연구 □ 기타( )		
**심사서류**	■ 신규연구계획 심의신청서<br>□ 연구계획 변경 심의신청서<br>□ 연구중간보고 심의신청서<br>□ 연구종료보고서<br>□ 동의서면화면제사유서<br>□ 동의면제사유서<br>■ 연구계획서 (Version: 1.0)<br>■ 연구진 생명윤리/연구윤리 관련 교육 이수증 사본<br>■ 연구책임자 이력서<br>■ 연구대상자 본인용 동의서 및 설명문 (Version: 1.0)<br>□ 법정대리인용 동의서 및 설명문 (Version :)<br>□ 증례기록서/실험일지/연구노트 등<br>□ 연구비 산정내역서<br>□ 피해보상규약<br>■ 연구대상자 모집 문건<br>□ 연구진 이해상충공개서<br>■ 설문지, 인터뷰/면담 질문지 등 연구 도구<br>□ 설문지 외에 연구대상자에게 제공되는 정보자료<br>□ 인간대상연구의 중재 관련 의약품/의료기기/화장품/기구/시술법/음식 등에 대한 정보 또는 자료<br>□ 인체유래물연구 관련 물질양도각서<br>□ 타기관 IRB 승인서<br>□ 심의의견에 대한 답변서<br>□ 변경대비표		

	■ 기타 (배포용 웹페이지)		
**심의결과**	■ 승인 □ 시정승인 □ 보완(재심의) □ 반려 □ 중지 또는 보류		
**연구승인기간**	2025.10.17.~2026.12.31. ※ 연구기간 연장 시 승인기간 만료 이전에 연구계획변경 신청 필요		
**심의 의견**	-		
**심의일**	2025.10.16.	**중간보고기한**	N/A

★ 모든 연구자들은 아래의 사항을 준수하여야 합니다.

- 울산과학기술원 생명윤리위원회는 생명윤리 및 안전에 관한 법률을 비롯한 연구 관련 국내법을 준수하고, 생명윤리 및 연구윤리 관련 국제규범과 국내규범을 존중합니다.
- 본 통지서에 기재된 내용은 울산과학기술원 생명윤리위원회에 기록 · 보관된 내용과 일치함을 증명합니다.
- 본 통지서의 사본은 울산과학기술원 생명윤리위원회에서 보관합니다.
- 위원회가 연구에 대해 조사하거나 감독하는 경우 적극 협조하여야 하고, 기타 위원회가 요구하는 경우 연구와 관련된 사항에 대하여 보고하여야 합니다.
- 시정승인 또는 보완에 대한 수정서류는 최초 결과 통지일로부터 2주 이내에 관련 서류와 함께 위원회로 제출해주시기 바랍니다.
- 본 통지서에 기재된 위원회의 심의 결과에 대하여 이의가 있는 경우 최초 결과 통지일로부터 2주 이내에 서면으로 이의신청을 할 수 있습니다. 단, 동일한 사안에 대하여 2회 이상 이의신청을 할 수 없습니다.
- 본 위원회에서 지정한 중간보고기한 최소 3주전까지 중간보고신청서를, 연구종료 후에는 3개월 이내에 종료보고서를 작성하여 제출해 주시기 바랍니다.
- 연구 중에 중대한 이상반응(Adverse Event) 발생 사항에 대해서는 위원회에 서면으로 보고하여 주시기 바랍니다.
- 연구 진행에 있어서 피험자를 보호하기 위한 불가피한 사항을 제외하고는 연구의 어떠한 변경이든 위원회의 사전승인을 받고 수행하여 주십시오.
- 인간을 대상으로 하는 연구 시, 반드시 본 위원회에서 승인 받은 피험자 동의서 및 설명문 양식만을 사용해야 합니다.
- 본 연구에 대한 위원회의 심의 결과는 위원회의 사전 승인 없이는 학술연구목적 이외의 목적으로 사용하지 않아야 합니다.
- 연구와 관련하여 다음과 같은 기록은 연구가 종료된 시점으로부터 최소 3년간 보관하여야 합니다.
  (1) 연구계획서 및 이에 대한 위원회의 심의 확인서
  (2) 연구대상자로부터 받은 서면동의서 또는 서면동의면제 승인서

(3) 개인정보의 수집·이용 및 제공 현황

(4) 연구 종료 보고서 및 연구 진행과정 및 결과에 대한 위원회의 조사·감독 결과

- 위원회 연락처 : 052)217-5214(줄기세포연구관, 109호), leeyj0926@unist.ac.kr

귀하가 신청한 위 연구과제에 대해

울산과학기술원 생명윤리위원회에서 위와 같이 결정하였음을 통지합니다.

2025. 10. 16.

UNIST 생명윤리위원회 위원장 강 병 헌 (인)

UNIST 생명윤리위원회위원장
