# Supplementary material for: Virtual reality exposure therapy with graded interviewer reactions for public speaking anxiety in university students: a randomized controlled trial protocol
Source: Trials. 2026 May 19;27:485. doi: 10.1186/s13063-026-09779-0 (PMC13352607; doi:10.1186/s13063-026-09779-0)
Supplement: Supplementary file 1 — Supplementary Material 1. [file 13063_2026_9779_MOESM1_ESM.zip › Supplementary5_Funding Agreement (Korean original with official signatures).pdf]

시점확인필
미검증
2024/04/04
08:21:35
KST
정부시점확인센터

# 표준협약서

(전문기관 및 주관연구기관 협약용)

| | | | |
|---|---|---|---|
| 연구개발사업명 | 과학기술인문융합연구사업 | | |
| 연구개발과제명 | (공동2)정신의학-예술 융합연구를 통한 불안증상 조절 디지털치료 콘텐츠 개발 및 실증 | | |
| 협약연구개발비 | 47,600 (단위:천원) | | |
| 부처 | 과학기술정보통신부 | 전문기관 | 한국연구재단 |
| 주관연구기관 | 울산과학기술원 총장 | 주관<br>연구책임자 | 김황 |

연구 개발비 (단위:천원)

| 구분 | 정부출연금 | 기업부담금 | 기타 | 계 |
|---|---|---|---|---|
| 1차 연도 | 75,000 | | | 75,000 |
| 2차 연도 | 84,000 | | | 84,000 |
| 3차 연도 | 47,600 | | | 47,600 |
| 4차 연도 | 110,000 | | | 110,000 |
| 5차 연도 | 100,000 | | | 100,000 |
| 6차 연도 | | | | |
| 7차 연도 | | | | |
| 8차 연도 | | | | |
| 계 | 416,600 | | | 416,600 |

| | |
|---|---|
| 총 연구개발 기간 | 2022년 06월 01일부터 2026년 12월 31일까지 |
| 다년도 협약 기간 | 2022년 06월 01일부터 2024년 12월 31일까지 |
| 당해연도 연구 기간 | 2024년 01월 01일부터 2024년 12월 31일까지 |

위 연구 개발 과제의 수행에 관하여 전문 기관과 주관 연구 기관 및 주관 연구 책임자는 다음의 협약 내용에 따라 협약을 체결한다.

2024년 04월

**[협약 당사자]**

전문기관 :
(과학기술정보통신부 장관 업무대행) 한국연구재단 이사장

주관연구개발기관 : 울산과학기술원 총장

주관연구책임자 : 김황

소 속 : 울산과학기술원 직급(위) : 부교수

첨부서류
1. 협동과제목록 1부(2개 이상의 세부과제로 구성된 경우에만 해당)
2. 연구개발비 집행계획서 1부 (2개 이상의 세부과제로 구성된 경우에만 해당)

제1조(목적) 이 협약은 「국가연구개발혁신법」 및 「국가연구개발혁신법 시행령」에 따라 연구개발과제의 수행에 필요한 연구개발계획, 중앙행정기관의 권한・의무, 전문기관의 권한・의무, 연구개발기관과 연구자의 권리・의무 등을 규정함을 목적으로 한다.

제2조(용어의 정의) 이 협약에서 사용하는 용어는 「국가연구개발혁신법」(이하 "법"이라 한다)과 「국가연구개발혁신법 시행령」(이하 "영"이라 한다)을 따른다.

제3조(연구개발계획서) 법 제11조제1항제1호에 따른 연구개발과제 수행 계획은 첨부서류1의 연구개발계획서에 따른다.

제4조(중앙행정기관의 권한과 의무) 중앙행정기관의 권한과 의무는 다음 각 호와 같다.
1. 연구개발과제 협약체결 및 수행에 관한 종합적인 지원
2. 연구개발비 중 정부지원금의 지급
3. 연구개발비의 관리 및 사용실적에 대한 점검, 정산
4. 연구개발과제에 대한 단계평가, 특별평가, 최종평가, 등 평가관리 및 후속조치
5. 연구개발기관 및 연구책임자에게 연구개발과제와 관련된 보고 또는 자료제출의 요구
6. 연구개발과제 성과의 활용에 대한 관리 및 감독
7. 연구개발과제 기술료의 징수 및 사용에 대한 관리
8. 연구개발정보의 수집, 관리 및 처리
9. 국가연구개발사업 정보에 대한 보안 관리 및 조치
10. 연구개발 지원체계, 확립 및 평가, 연구개발 관련 교육・훈련
11. 연구개발기관에 대한 제도개선의 권고 및 감독
12. 연구윤리 확보 및 제재처분 등 연구개발과제의 성실수행을 위한 조치
13. 그 밖에 제1호부터 제12호까지의 업무를 수행하는데 필요한 부수적인 업무, 기타 관련 규정에서 정한 사항

제5조(전문기관의 권한과 의무) 전문기관은 제4조에서 정하는 중앙행정기관의 권한과 의무를 대행한다.

제6조(연구개발기관의 권리와 의무) ① 주관연구개발기관의 권리와 의무는 다음 각 호와 같다.
1. 연구개발과제 협약 체결 및 수행에 대한 종합적인 관리
2. 연구개발과제 수행 총괄 및 다른 참여 연구개발기관과의 협력
3. 연구개발계획서의 사전 검토・조정 및 작성 총괄
4. 주관연구개발기관이 부담하기로 한 연구개발비의 부담
5. 연구개발비의 사용・관리 및 사용실적 보고 총괄
6. 연구개발과제 수행에 필요한 연구자, 시설의 확보 및 연구지원
7. 연구개발과제의 연차보고서・단계보고서・최종보고서 등 각종 보고서 작성 총괄
8. 연구개발성과의 활용 및 성과활용보고서 등 작성 총괄
9. 기술료의 징수・사용, 기술료의 일부 또는 연구성과로 인한 수익의 일부 납부 및 징수 실적의 보고
10. 「과학기술기본법」 제12조제1항에 따른 국가연구개발사업의 조사・분석・평가에 필요한 자료 제출
11. 연구개발과제의 보안관리
12. 연구윤리규정 마련 및 운영
13. 연구시설・장비의 활용, 관리 및 관련 자료의 제공
14. 부정행위 등 발생 시 중앙행정기관에 보고
15. 연구노트의 작성・관리에 관한 자체지침 마련 및 관리
16. 그 밖에 법 또는 영에서 주관연구개발기관의 권한과 책임으로 정한 사항
② 주관연구개발기관이 연관되어 추진되는 연구개발과제들을 총괄하는 경우에 그에 따른 권리와 의무는 다음 각 호

와 같다.
1. 연관되어 추진되는 연구개발과제들의 총괄관리
2. 연관되어 추진되는 연구개발과제들의 연구개발계획서 사전 검토ㆍ조정 및 전체 연구계획 수립
③ 공동연구개발기관의 권리와 의무는 다음 각 호와 같다.
1. 연구개발과제 협약 체결 및 수행에 대한 관리
2. 연구개발과제에 공동 참여 및 다른 참여 연구개발기관과의 협력
3. 연구개발계획서의 작성
4. 공동연구개발기관이 부담하기로 한 연구개발비의 부담
5. 연구개발과제 수행에 필요한 연구자, 시설의 확보 및 연구지원
6. 연구개발비의 사용ㆍ관리 및 사용실적 보고
7. 연구개발과제의 연차보고서ㆍ단계보고서ㆍ최종보고서 등 각종 보고서 작성
8. 연구개발성과의 활용 및 성과활용보고서 등 작성
9. 기술료의 징수ㆍ사용, 기술료의 일부 또는 연구성과로 인한 수익의 일부 납부 및 징수 실적의 보고
10. 「과학기술기본법」 제12조제1항에 따른 국가연구개발사업의 조사ㆍ분석ㆍ평가에 필요한 자료 제출 협조
11. 연구개발과제의 보안관리
12. 연구윤리규정 마련 및 운영
13. 연구시설ㆍ장비의 활용, 관리 및 관련 자료의 제공
14. 부정행위 등 발생 시 중앙행정기관에 보고
15. 연구노트의 작성ㆍ관리에 관한 자체지침 마련 및 관리
16. 그 밖에 법 또는 영에서 공동연구개발기관의 권한과 책임으로 정한 사항
④ 위탁연구개발기관의 권리와 의무는 다음 각 호와 같다.
1. 연구개발과제 협약 체결 및 수행에 대한 관리
2. 연구개발과제에 공동 참여 및 다른 참여 연구개발기관과의 협력
3. 연구개발계획서의 작성
4. 연구개발과제 수행에 필요한 연구자, 시설의 확보 및 연구지원
5. 연구개발비의 사용ㆍ관리 및 사용실적 보고
6. 연구개발과제의 연차보고서ㆍ단계보고서ㆍ최종보고서 등 각종 보고서 작성
7. 기술료의 징수ㆍ사용, 기술료의 일부 또는 연구성과로 인한 수익의 일부 납부 및 징수 실적의 보고
8. 「과학기술기본법」 제12조제1항에 따른 국가연구개발사업의 조사ㆍ분석ㆍ평가에 필요한 자료 제출 협조
9. 연구개발과제의 보안관리
10. 연구윤리규정 마련 및 운영
11. 연구시설ㆍ장비의 활용, 관리 및 관련 자료의 제공
12. 부정행위 등 발생 시 중앙행정기관에 보고
13. 연구노트의 작성ㆍ관리에 관한 자체지침 마련 및 관리
14. 그 밖에 법 또는 영에서 공동연구개발기관의 권한과 책임으로 정한 사항

제7조(연구자의 권리와 의무) ① 연구자의 권리와 의무는 다음 각 호와 같다.
1. 자율과 책임을 바탕으로 성실하게 국가연구개발과제를 수행해야 한다.
2. 국가연구개발과제를 수행함에 있어 도전적으로 자신의 능력과 창의력을 발휘하되, 경제적ㆍ사회적 영향을 고려해야 한다.
3. 연구윤리를 준수하고 진실하고 투명하게 국가연구개발과제를 수행해야 한다.
② 연구책임자는 연구개발에 참여하는 연구자가 연구개발 활동에 전념할 수 있도록 배려해야 한다.

제8조(협약의 변경) 이 협약의 변경에 관하여는 법 제11조제2항과 영 제14조에 따른다.

제9조(협약의 해약) 이 협약의 해약에 관하여는 법 제11조제4항과 영 제15조에 따른다.

제10조(연구개발결과 보고) 연차보고서, 단계보고서, 최종보고서, 성과활용보고서의 제출에 관하여는 법 제12조제4항부터 제6항까지의 규정과 영 제18조에 따른다.

제11조(연구개발과제의 평가) ① 연구개발과제의 단계평가, 최종평가에 관하여는 법 제12조제2항, 제14조 및 제15조와 영 제16조 및 제25조부터 제29조까지의 규정에 따른다.
② 중앙행정기관의 장은 필요한 경우에 구체적인 평가 기준・방법・절차 등을 정하여 단계평가, 최종평가, 특별평가를 실시할 수 있다.

제12조(평가에 따른 조치) 단계평가, 최종평가, 특별평가 결과에 따른 조치에 관하여는 법 제12조제3항 및 제15조와 영 제17조에 따른다.

제13조(연구개발비 부담・지급・사용・관리 및 사용내역 보고・정산 등) ① 중앙행정기관의 장은 정부지원금을 다음과 같이 연구개발기관에 지급해야 한다.

| | | |
|---|---|---|
| (가) 제 1 차 : | 2024년 01월 일 | 47,600 천원 |
| (나) 제 2 차 : | 년 월 일 | 천원 |
| (다) 제 3 차 : | 년 월 일 | 천원 |
| (라) 제 4 차 : | 년 월 일 | 천원 |
| (마) 제 5 차 : | 년 월 일 | 천원 |
| (라) 제 6 차 : | 년 월 일 | 천원 |
| (라) 제 7 차 : | 년 월 일 | 천원 |
| (라) 제 8 차 : | 년 월 일 | 천원 |
| (라) 제 9 차 : | 년 월 일 | 천원 |
| (라) 제 10 차 : | 년 월 일 | 천원 |
| (라) 제 11 차 : | 년 월 일 | 천원 |
| (마) 제 12 차 : | 년 월 일 | 천원 |

② 연구개발기관의 장은 부담하기로 한 연구개발비 중 현금을 중앙행정기관이 지정하는 계좌로 입금해야 한다.
③ 제1항에도 불구하고 다음 각 호의 경우에 국가연구개발사업의 추진목적・성격 등을 고려하여 정부지원금의 지급 횟수, 시기, 지급 조건・방법 등을 조정할 수 있다.
1. 연구개발비의 절감 또는 연구개발기간 단축 등에 따라 협약이 변경되는 경우
2. 정부의 예산 사정, 관련 법령 또는 규정의 개정이나 정부의 정책 변경 등으로 인하여 정부지원금의 조정이 필요한 경우
3. 본 협약이 변경 또는 해약되는 경우
4. 중앙행정기관의 장이 지정하는 자의 현장실태조사 또는 평가 등의 결과에 따라 조정이 필요한 경우
5. 기타 중앙행정기관의 장의 통제범위를 초월하는 사태의 발생으로 조정이 필요한 경우
④ 연구개발기관의 장은 다음 각 호의 어느 하나의 방법으로 영 제24조에 따른 연구개발비카드(이하 "연구개발비카드"라 한다)를 발급받거나 지정해야 한다.
1. 통합정보시스템 도입을 통하여 해당 연구개발과제와 연결된 연구개발비카드를 신청하여 발급
2. 결제계좌가 연구개발기관의 법인계좌인 법인카드를 통합정보시스템에 등록하여 해당 법인카드를 연구개발비카

드로 지정
⑤ 중앙행정기관의 장은 연구개발기관이 연구개발비를 부당 또는 과다하게 책정하였음을 발견할 때에는 다음 각 호의 조치를 취할 수 있다.
1. 발견시점이 연구개발과제 협약기간 중인 경우에는 부당 또는 과다하게 책정한 금액을 감액 또는 회수
2. 발견시점이 연구개발과제 협약기간 종료 후, 정산 전인 경우에는 부당 또는 과다하게 책정한 금액을 정산 시 불인정하여 회수
3. 발견시점이 정산 후인 경우에는 재정산을 실시하여 부당 또는 과다하게 책정한 금액을 불인정하여 회수
⑥ 제1항부터 제5항까지에서 정한 사항 외에 연구개발비 부담・지급・사용・관리 및 사용내역 보고・정산 등에 관하여는 법 제13조, 영 제19조부터 제26조까지의 규정과 법 제13조 또는 영 제19조부터 제26조까지의 규정에서 위임한 사항을 정하는 행정규칙에서 정하는 바에 따른다.

제14조(연구개발성과의 소유) 연구개발성과의 소유에 관하여는 법 제16조와 영 제32조에 따른다.

제15조(연구개발성과의 관리) 연구개발성과의 관리에 관하여는 법 제16조제4항과 영 제33조에 따른다.

제16조(연구개발성과의 활용・공개) ① 연구개발기관은 연구개발과제의 수행 과정 및 결과에 대한 홍보, 공개 또는 발표 등을 할 경우에 다음 각 호에 따른 필수 사항을 밝혀야 한다.
1. 연구개발성과가 논문인 경우: 정부의 연구개발비 지원 연도, 연구개발비를 지원한 중앙행정기관명, 연구개발과제번호, 연구개발과제명, 연구개발과제별 기여율(연구개발과제가 2개 이상인 경우에 해당되며, 각 기여율의 합은 100퍼센트가 되도록 한다)
2. 연구개발성과가 특허인 경우: 연구개발비를 지원한 중앙행정기관명, 국가연구개발사업명, 연구개발과제번호, 연구개발과제명, 연구개발과제별 기여율(연구개발과제가 2개 이상인 경우에 해당되며, 각 기여율의 합은 100퍼센트가 되도록 한다)연구개발과제의 연구개발기간
3. 연구개발성과를 언론에 홍보하는 경우: 정부의 연구개발비 지원 연도 및 금액, 연구개발비를 지원한 중앙행정기관명, 국가연구개발사업명
② 연구개발기관 또는 연구개발기관 소속 임직원이 연구개발성과를 개량하여 새로운 성과(이하 "개량성과"라 한다)를 창출하거나 지식재산권 등을 취득하였을 경우에 개량성과의 실시에 관한 계약은 연구개발성과의 실시에 관한 계약으로 본다.
③ 연구개발기관은 보완된 최종보고서를 제출한 후 3개월 이내에 최종보고서와 등록・기탁한 연구개발성과 목록을 통합정보시스템을 통하여 공개해야 한다. 다만, 비공개 특허는 공개하지 않을 수 있다.
④ 그밖에 연구개발성과의 활용・공개에 관하여는 법 제17조와 영 제34조・제35조에 따른다.

제17조(기술료 등의 징수・납부・사용) 기술료의 징수・감면・사용, 기술료 또는 연구개발성과로 인한 수익의 납부・감면에 관하여는 법 제18조, 영 제38조부터 제41조까지의 규정과 법 제18조 또는 영 제38조부터 제41조까지의 규정에 따라 중앙행정기관이 정하는 바에 따른다.
② 기술료등납부의무기관의 연구개발성과로 인한 수익의 납부와 관련하여 영 제39조제2항에 따른 기술기여도는 기술자산이 이익창출에 기여한 상대적인 공헌도에 따라 정한다.

제18조(연구시설・장비의 등록・관리) 연구시설・장비의 등록・관리에 관하여는 「과학기술기본법」 제28조와 「과학기술기본법」 제28조에서 위임한 사항을 정하는 행정규칙에서 정하는 바에 따른다.

제19조(연구개발정보의 처리 등) ① 연구개발기관의 장과 연구책임자는 중앙행정기관의 장이 지정하는 자의 연구개발 현장 확인, 관계 서류의 열람, 관계 자료의 제출요청 등에 성실히 응해야 한다.
② 이 협약에 따른 어느 하나의 당사자는 관련 자료를 다른 당사자에게 통지할 때 이 협약에 따른 다른 당사자의 주소로 내용증명, 등기우편 또는 전자문서 등 서면으로 해야 한다. 이 경우 전자문서는 다음 각 호의 어느 하나의 방법

으로 해야 한다.
1. 연구개발기관이 통합정보시스템에 등록한 전자메일 주소를 통한 전송
2. 통합정보시스템을 통한 전자문서 발송
3. 협약 당사자의 정보시스템을 통한 전자문서 발송
③ 이 협약에 따른 어느 하나의 당사자는 주소가 변경되는 경우에 즉시 나머지 당사자에게 이를 통지해야 한다.
④ 그 밖에 연구개발정보의 처리 등에 관하여는 법 제19조・제20조, 영 제42조・제43조와 법 제19조・제20조 또는 영 제42조・제43조에서 위임한 사항을 정하는 행정규칙에서 정하는 바에 따른다.

제20조(연구실 등의 안전관리) ① 연구개발기관은 「연구실 안전환경 조성에 관한 법률」 및 「산업안전보건법」 등 관련 법령에 따른 연구실 안전 관련 사항 또는 연구개발기관의 연구실 안전관리규정에서 정하는 사항을 준수해야 한다.
② 연구개발기관이 공공기관인 경우에 안전관리에 관하여는 「공공기관의 운영에 관한 법률」에서 위임한 사항을 정하는 지침에서 정하는 바에 따른다.
제21조(보안관리) ① 연구개발기관의 장은 영 제44조제3항에 따라 이 협약을 체결한 날부터 1개월 이내에 연구보안담당자를 지정하고 보안관리규정을 마련하여 운영해야 한다.
② 제1항에서 정한 사항 외에 보안관리에 관하여는 법 제21조와 영 제44조부터 제48조에 따른다.

제22조(연구지원체계 확립 등) 연구지원체계의 확립 및 소속 연구자・연구지원인력의 역량 강화에 관하여는 법 제24조부터 제26조까지, 영 제51조・제52조와 법 제24조부터 제26조까지 또는 영 제51조・제52조에서 위임한 사항을 정하는 행정규칙에서 정하는 바에 따른다.

제23조(부정행위 등에 대한 제재처분 등) 부정행위 등의 제재처분과 연구개발비 환수에 관하여는 법 제31조부터 제34조까지, 영 제56조・제57조, 제59조부터 제63조까지, 「국가연구개발혁신법 시행규칙」(이하 "규칙"이라 한다)과 연구개발기관의 부정행위의 검증・조치・보고에 관한 자체규정에 따른다.

제24조(연구노트 작성・관리) 연구노트 작성・관리에 관하여는 법 제35조제2항, 영 제65조제1항 및「국가연구개발사업 연구노트 지침」에 따른다.

제25조(동시수행 연구개발과제 수 관리) 연구개발기관의 장은 소속 연구자가 연구에 전념할 수 있도록 배려해야 하며, 소속 연구자가 영 제64조에 따른 연구자가 동시에 수행할 수 있는 연구개발과제의 수 제한을 준수하도록 관리해야 한다.

제26조(관계법령등의 준수) ① 연구개발기관의 장은 연구개발과제를 수행함에 있어서 이 협약에 포함되지 않는 사항에 관하여 법, 영, 규칙, 법・영 또는 규칙에서 위임한 사항을 규정하는 행정규칙과 해당 국가연구개발사업의 추진 근거가 되는 법령, 법령에서 위임한 사항을 규정하는 행정규칙(이하 "관계법령등"이라 한다)을 준수해야 한다.
② 법, 영, 규칙, 법・영 또는 규칙의 해석에 관하여는 과학기술정보통신부장관의 해석에, 해당 국가연구개발사업의 추진 근거 법령, 법령에서 위임한 사항을 규정하는 행정규칙의 해석에 관한 사항은 소관 중앙행정기관의 장의 해석에 따른다.

제27조(부가조건) ① 본 협약의 조항에도 불구하고 본 협약의 목적 달성을 위해 전문기관의 장이 주관연구기관의 장을 경유하여 주관연구책임자에게 본 협약 체결에 앞서 미리 지급한 연구개발비가 있는 경우 동 금액은 본 협약 제13조에 의한 연구개발비의 지급으로 본다.
② 주관연구기관의 장은 주관연구책임자에게 제1항에 의해 선지급된 연구개발비가 본 협약에서 정한 바에 따라 지출되었음을 진술 및 보증하며 선지급하되 주관연구책임자는 연구개발비의 목적외 사용, 기타 협약 위반 등의 사유가 발견되는 경우 본 협약에서 정한 바에 따른 제재, 연구개발비 회수 조항이 적용되는 것을 인지하고 있으며 동 조항의

적용에 동의한다.
③ 연구비통합관리시스템(통합이지바로)의 연구수행기관별 연구개발비는 아래 각 호의 기준으로 지급한다.
1. 일괄지급 : 대학, 출연(연), 특정(연), 테크노파크, 병원 등 비영리법인, 정부(지자체), 공기업 및 외국 소재의 기관
2. 건별지급 : 기업(대기업, 중견기업, 중소기업, 개인사업자) 및 학회, 협회, 병원 등 비영리법인 중 연구비 관리 전담부서가 없는 기관
④ 주관연구기관의 장은 처리규정 제27조(연구개발비 사용내역 관리)에 따라 「연구지원시스템 통합 추진단 설치 및 운영에 관한 규정」에서 정하는 바에 따라 다음 각 호에서 정한 기한 내에 연구개발비 사용 내역 등을 연구비통합관리시스템에 입력하여야 하며, 이를 시행하지 않는 경우 불이익한 처분을 받을 수 있다.
1. 일괄지급 대상 기관 : 집행 후 5일 이내
2. 건별지급 대상 기관 : 연구비 사용내역 입력 후 집행
⑤ 전문기관의 장은 주관연구책임자가 과제 수행 중에 기초과학연구원 단장, 그룹리더 등 타 사업으로의 이동을 원칙적으로 불인정하고, 위반 시 과학기술기본법, 국가연구개발사업혁신법, 과학기술정보통신부 소관 연구개발사업 처리규정 등에 따라 정당한 사유 없이 연구개발과제의 수행을 포기한 불성실한 연구로 간주하여 참여제한 및 지급 연구비를 전액 환수할 수 있다. 단, 연구과제가 연구 종료(단계 종료) 예정인 경우, 종료 시점에 연구목표를 달성하고 이동하려는 사업과 연계성이 있다고 검증되는 경우에는 중단을 허용할 수 있다.
⑥ 전문기관의 장은 협약기간 또는 협약 종료 후 본 표준협약서 및 관련 규정에 명시된 연구과제 관련 주요사항 보고 및 관계자료 제출을 요청할 수 있으며, 주관연구기관의 장은 이에 응해야 한다.
⑦ 연구개발과제를 수행하는 연구책임자 및 공동연구원은 전문기관의 장이 타 연구과제의 평가위원으로 참여를 요청 시, 적극 협조하여야 한다.
⑧ 참여연구원 중에 학생연구원이 있는 경우 영 제41조제3항에 따른 기술료의 사용에 관한 사항에 대해 학생연구원 보상금 지급 기준, 지급 절차 등을 마련하고 그 기준에 따라 지급하여야 한다.
⑨ 각 연차의 연구기간 및 연구비는 제10조에 따라 연차별로 제출하는 연차보고서의 당해 연도 연구기간 및 연구비와 동일하며, 사업 수행이나 관리 시 연차별로 다른 기준을 적용해야 하는 경우 각 연차의 연구기간과 연구비를 적용한다.
⑩ (특약사항)기타 사업 운영 관련 사항은 상위 규정 및 전문기관 사업관리지침에 따르도록 한다.
⑪ (업무대행)본 협약과 관련하여 법 제22조 제1항에 따라 전문기관의 장은 중앙행정기관(과기정통부)의 장의 업무를 대행한다.
⑫ 전문기관과 연구개발기관과의 일괄협약 체결을 위하여 통합정보시스템 구축 후 다시 협약을 체결할 수 있다.
⑬ 주관연구개발기관이 공동연구개발기관과 함께 연구를 수행하는 경우, 해당 공동연구개발 과제는 법령에 불구하고, 연구사업통합지원시스템(e-R&D)상 “단위과제”의 형태로 관리될 수 있다.
⑭ 공동연구개발과제가 포함된 주관연구개발과제의 연구비는 공동연구개발과제 연구비를 제외한 금액으로 표기되며, 전문기관은 통합이지바로시스템을 통해 공동연구개발기관과 주관연구개발기관에 각각의 연구비를 별도 지급할 수 있다.

2024년 04월

**전문기관 :**
**(과학기술정보통신부 장관 업무대행)** **한국연구재단 이사장**

**주관연구개발기관 :** **울산과학기술원 총장**

주관연구책임자

소속 : 울산과학기술원 직급(위) : 부교수 성명 : 김황

첨 부 : 1.협동과제목록 1부(2개 이상의 세부과제로 구성된 경우에만 해당)

2 연구개발비 집행계획서 1부 (2개 이상의 세부과제로 구성된 경우에만 해당)

[첨부 1] 협동과제목록

| 연구과제명 | 연구개발비(천원) | | | | 연구시작 | 주관연구기관명 |
|---|---|---|---|---|---|---|
| | 정부 | 기업 | 기타 | 계 | 연구종료 | 연구책임자명 |
| 조회된 데이터가 없습니다. | | | | | | |

[첨부 2] 연구비 집행계획서

(단위 : 천원)

| 지급시기 / 과제명 | 1차 | 2차 | 3차 | 4차 | 5차 | 6차 |
|---|---|---|---|---|---|---|
| | (2024.01) | (　.　) | (　.　) | (　.　) | (　.　) | (　.　) |
| (공동2)정신의학-예술 융합연구를 통한 불안증상 조절 디지털치료 콘텐츠 개발 및 실증 | 47,600 | | | | | |

| 지급시기 / 과제명 | 7차 | 8차 | 9차 | 10차 | 11차 | 12차 |
| --- | --- | --- | --- | --- | --- | --- |
| | ( . ) | ( . ) | ( . ) | ( . ) | ( . ) | ( . ) |
| (공동2)정신의학-예술 융합연구를 통한 불안증상 조절 디지털치료 콘텐츠 개발 및 실증 | | | | | | |
