# Supplementary material for: Virtual reality exposure therapy with graded interviewer reactions for public speaking anxiety in university students: a randomized controlled trial protocol
Source: Trials. 2026 May 19;27:485. doi: 10.1186/s13063-026-09779-0 (PMC13352607; doi:10.1186/s13063-026-09779-0)
Supplement: Supplementary file 1 — Supplementary Material 1. [file 13063_2026_9779_MOESM1_ESM.zip › Supplementary6_Annual Research Progress Report 2024 Summary (Korean original).pdf]

시점확인 진본 2025/01/31 11:01:16 KST 정부시점확인센터

2024년도 STEAM연구사업 단계보고서 및 계획서(평가용(IRIS), 주관과제용)

양식A101-3

① 부처사업명(대)	원천기술개발사업	④ 보안등급(보안, 일반)	일반
② 사업명(중)	STEAM연구사업	⑤ 과제성격(기초, 응용, 개발)	개발
③ 세부사업명(소)	과학기술인문융합연구사업		

⑥ 단위과제명	국 문	정신의학-예술 융합연구를 통한 불안증상 조절 디지털치료 콘텐츠 개발 및 실증
	영 문	Development and verification of digital therapy contents for anxiety through psychiatry-art convergence research

⑦ 주관연구기관명	고려대학교		⑧ 사업자 등록번호	2098208298
⑨ 위탁과제기관명				
⑩ 주관연구책임자	성 명	조철현	국가연구자번호	11192235
	전 공	정신과학	직급(직위)	교수
	소속부서	고려대학교 의과대학	전자우편	david0203@gmail.com
	전 화	010-9167-2203	휴대전화	010-9167-2203

⑪ 연구개발비 현황(단위: 천원)

년 도	정부 출연금 (A)	기업체부담금			정부외출연금 (B)	합계 G=(A+B+E)	상대국 부담금 (F)
		현금 (C)	현물 (D)	소계 E=(C+D)			
1차년도	420,000	4,200	37,800	42,000		462,000	
2차년도	600,000	6,000	54,000	60,000		660,000	
3차년도				0		0	
4차년도				0		0	
5차년도				0		0	
6차년도				0		0	
합계	1,020,000	10,200	91,800	102,000	0	1,122,000	0

⑫ 총연구기간	2023. 07. 01 - 2027. 12. 31 (54개월)
⑬ 다년도연구기간	2024. 01. 01 - 2025. 12. 31 (24개월)
⑭ 당해연도연구기간	2024. 01. 01 - 2024. 12. 31 (12개월)

⑮ 참여기업 수	중소기업	1	중견기업		대기업		계	
⑯ 국제공동연구	국가명		상대국 연구기관수		상대국 연구개발비		상대국연구책임자수	
⑰ 실무담당자	성 명	윤소진	휴대전화	010-4222-2490	전자우편	illuminityy@naver.com		

관련 법령 및 규정과 모든 지시 시항을 준수하면서 이 국가연구개발사업을 성실히 수행하고자 아래와 같이 연구개발계획서(연구개발제안서)를 제출합니다. 아울러 이 연구개발계획서(연구개발제안서)에 기재된 내용이 사실임을 확인하며, 만약 사실이 아닌 경우 선정 취소, 협약 해약 등의 불이익도 감수하겠습니다.

주관연구책임자 : (직인생략)

주관연구기관장 : (직인생략)

과 학 기 술 정 보 통 신 부 장 관 귀하

ㅇ 공동연구개발기관 등 현황 (해당시 작성)

공동연구개발기관 등 (해당 시 작성)	기관명	책임자	직위	휴대전화	전자우편	비고	
						역할	기관유형
공동연구개발기관	한국예술종합학교	조충연	교수	010-3317-0442	yellowsub@karts.ac.kr	*공동책임*	*대학*
	울산과학기술원	김황	부교수	010-4719-5226	hwangkim@unist.ac.kr	*공동책임*	*특정연구기관(기타공공기관)*
	(주)메디마인드	김남석	부장	010-3064-2178	gtferrari@medimind.kr	*공동책임*	*중소기업*
위탁연구개발기관							
연구개발기관 외 기관							

〈 요 약 문 〉

	양식A201
**연구개발 목표 (500자 내외)**	현대인이 보편적으로 경험하는 불안의 세부증상과 중증도는 다양하게 나타남. 세부증상의 특성에 따라 적용하는 정신치료 기법은 상이하고 증상호전이 제한적임. 불안증상별 정신치료 기법을 VR 디지털치료에 적용할 수 있는 정신심리-예술-공학-산업 융합연구팀을 구성하여 각 기관별 특장점을 발휘해 불안조절 VR 디지털치료 콘텐츠 시제품을 개발을 목표로 세움. 더 나아가 불안증상 조절 VR 디지털치료를 이용하여 효과 검증을 위한 임상연구 및 상용화를 목표로 함.
**연구개발 내용 (1000자 내외)**	불안의 세부 증상에 대한 다양한 정신치료적 기법을 정신의학-예술 융합 기반 VR 디지털치료 콘텐츠 개발 및 실증, 상용화를 목표로 함.<br>- 세부 불안 증상(부동불안, 공황불안-광장공포, 사회공포-수행불안)을 세분화하여 치료 카테고리를 분류하고, 각 증상에 대한 선행연구 기반 정신치료 기법 선별<br>- VR 디지털 치료 특성 및 효과 분석하여 최종 정신치료 기법을 확정 및 예술-기술적 측면을 고려한 정신치료 트랜스포밍<br>- 불안조절 VR 디지털치료를 위한 정신의학-예술 융합 기반 치료 콘텐츠 제작 (불안 증상별 시나리오, 실감콘텐츠 및 메타휴먼 등), 사용자 경험 및 서비스 디자인 적용, 그리고 시제품 개발, 불안조절 VR 디지털치료 콘텐츠 활용 치료 매뉴얼 및 프로토콜 개발<br>- 불안조절 VR 디지털치료 콘텐츠 효과 검증을 위해 임상연구 및 분산형 추적연구로 리얼월드데이터 확보<br>- 정신의학-예술 융합 불안조절 VR 디지털치료 콘텐츠 및 UX/UI 고도화 및 테스트베드 적용 및 리얼월드데이터 확보<br>- 불안조절 VR 디지털치료 콘텐츠 메타버스 상용화 전략 수립

활용계획 및 기대효과 (500자 내외) (응용분야 및 활용범위 포함)	- 개인맞춤형 디지털치료를 적용하여 불안 뿐 아니라 기분 및 스트레스 관리 등에 확대 적용할 수 있겠음.- 메타버스 기반 VR 디지털치료 콘텐츠 적용을 통해 준임상군-임상군의 불안 증상을 개선하는 효과를 기대함. 특히, 세부 불안증상별 적합한 디지털치료로 구성된 본 연구의 성과물은 증상별, 중증도별, 상황별 개인맞춤형 치료 옵션을 제공할 수 있을 것으로 기대됨.<br>- 정신의학-예술-기술 융합으로 사용자 디자인 공학 적용을 통해 사용성 개선에 따라, 치료효과 및 치료에 대한 만족도, 그리고 치료 순응도 향상을 기대함.<br>- 메타버스 플랫폼과의 연동을 동한 VR 디지털치료 제품 보급 및 산업적 수익 증대를 기대해볼 수 있겠음.<br>- 공공, 산업, 교육 등 지역사회 및 기관 내 여러 영역에서 직장 내 스트레스 및 직원 안전 등에 정신건강 관리가 포함되어 디지털치료가 보다 손쉽게 제공될 수 있는 정신건강 치료 플랫폼으로 제공될 수 있겠음.<br>- 과학기술적 측면: 메타버스 기반 디지털치료 기술 및 콘텐츠 개발을 통해 불안 증상 진단평가 및 치료 패러다임의 변화를 기대. AR/VR 기반 디지털 치료의 활용범위 확대로 기존의 특정 공포증이나 외상후스트레스장애 뿐만 아니라 물질장애, 주의력결핍과잉행동장애, 자폐, 사회기술훈련 등 다양한 정신건강 분야로의 확대가 예상됨. 사용자-친화적 휴먼감성 디지털 치료의 근거 기반 개발로, 블안증상과 같은 특정 상황에 대응 가능한 상호작용 방식을 개발하여 User-centered DTx design의 글로벌 기준을 제공할 수 있을 것으로 기대됨.<br>- 메타버스 기반 불안증상 디지털체료제 개발 및 보급을 통한 국민 정신건강 증진을 통한 경제적 비용 절감과 현대인들의 정신건강 서비스 진입장벽을 감소시킬 것을 기대함.			
국문핵심어 (8개)	불안	가상현실	디지털치료	미디어아트
	사용자 경험	디지털 실감 콘텐츠	이머시브 스토리텔링	상용화
영문핵심어 (8개)	Anxiety	Virtual Reality	Digital Therapy	Media Art
	User Experience	Digital Real Contents	Immersive Storytelling	Commercialization

ㅇ 단계별 목표 · 현재 기술수준(TRL)

기술수준(TRL)	1단계 목표	시 제 품 제작	2단계 목표	임상시험 및 상용화	3단계 목표		현재	

ㅇ 단계별 연구개발 목표와 내용

연구개발<br>목표 및 내용	1단계	목표	정신의학-예술 융합 기반 불안증상별 정신치료 적용 VR 디지털치료 콘텐츠 시제품 제작
		내용	- 선행연구 기반 불안증상별 정신치료 기법 트랜스포밍 및 타겟팅 세부 불안증상 분류화<br>- 불안조절 VR 디지털치료를 위한 정신심리-예술 융합 기반 치료 콘텐츠 (시나리오, 실감콘텐츠, 메타휴먼 등) 제작<br>- 불안 메타버스기반 VR 디지털치료 콘텐츠, 사용자 경험 및 서비스 디자인 적용<br>- 불안 조절 VR 디지털치료 콘텐츠 시제품 개발
	2단계	목표	불안조절 VR 디지털치료 콘텐츠 효과 검증 임상연구 수행 및 상용화
		내용	- 디지털표현형 기반 불안증상 조절 VR 디지털치료 효과 탐색 임상연구 및 분산형 추적연구로 리얼월드데이터 확보<br>- 정신의학-예술 융합 불안조절 VR 디지털치료 콘텐츠 및 UX/UI 고도화<br>- 불안조절 VR 디지털치료 콘텐츠 실증 지원 및 사용자 경험 디자인 및 인터페이스 완성, 테스트베드 적용 및 리얼월드데이터 확보<br>- 불안조절 VR 디지털치료 콘텐츠 메타버스 연동 타진 및 상용화 전략 수립

〈 목　차 〉

Ⅰ. 전 단계 연구개발 실적 ……………………………………………………

1. 연구개발 목표 및 평가항목별 성과 ……………………………………

2. 추진내용 및 연구개발결과 ……………………………………………

Ⅱ. 차기단계 연구개발 계획 ……………………………………………………

1. 연구개발 목표 및 평가항목 ……………………………………………

2. 연구개발 내용·방법, 추진체계 및 일정 ………………………………

3. 참여인력 계획 ……………………………………………………………

4. 연구개발비 집행계획 ……………………………………………………

5. 연구개발 성과의 활용방안 및 기대효과 ………………………………

6. 사업화 추진 계획 …………………………………………………………

Ⅲ. 당초 연구계획 대비 주요 변경사항 ………………………………………

1. 전 단계 실적 부분 …………………………………………………………

2. 차기단계 계획 부분 ………………………………………………………

첨부1 연구개발목표의 달성도 증빙 ……………………………………………

첨부2 연구데이터 관리계획(DMP) ……………………………………………

첨부3 [해당 시] 연구장비도입 심의요청서 ……………………………………

첨부4 [협약용 계획서 제출 시(해당 시)] 청년 의무채용 관련 실적 …………

첨부5 [협약용 계획서 제출 시] 평가의견에 대한 수정 · 보완 대비표 …………

첨부6 [협약용 계획서 제출 시] 안전관리 계획 등 ……………………………

첨부7 [해당 시] 영리기관의 연구실운영비 활용관리 계획 ……………………
