# Supplementary material for: Virtual reality exposure therapy with graded interviewer reactions for public speaking anxiety in university students: a randomized controlled trial protocol
Source: Trials. 2026 May 19;27:485. doi: 10.1186/s13063-026-09779-0 (PMC13352607; doi:10.1186/s13063-026-09779-0)
Supplement: Supplementary file 1 — Supplementary Material 1. [file 13063_2026_9779_MOESM1_ESM.zip › Supplementary8_Participant Information and Consent Form (Korean original with offical seal).pdf]

연구대상자 동의설명문

Version : 1.0

*동의설명문 변경 시 반드시 버전을 업그레이드하여 표기하여야 함.

<table>
<tr><th colspan="5">기본정보</th></tr>
<tr><td>IRB 승인번호</td><td colspan="4">피험자에게 제공 시 IRB 승인번호를 기입하여 사용</td></tr>
<tr><td rowspan="2">연구제목</td><td colspan="4">(국문) 사회평가적 위험 상황에서의 가상현실 면접 노출치료가 발표불안에 미치는 효과: 상황적 요소와 상호작용 요소를 고려한 무작위 대조 시험</td></tr>
<tr><td colspan="4">(영문) Effects of Virtual Reality Interview Exposure Therapy on Public Speaking Anxiety in Social-Evaluative Threat Situations: A Randomized Controlled Trial Considering Situational and Interactive Factors</td></tr>
<tr><td rowspan="4">연구책임자</td><td>성 명</td><td>소 속</td><td>직 위</td><td>전공분야</td></tr>
<tr><td>정두영</td><td>의과학대학원</td><td>부교수</td><td>정신의학</td></tr>
<tr><td>전화번호</td><td>팩스</td><td colspan="2">이메일</td></tr>
<tr><td>010 9797 0807</td><td>-</td><td colspan="2">dooyoung@unist.ac.kr</td></tr>
<tr><td colspan="5">※본 연구에 대한 문의사항이 있으시거나 동의서에 표기된 위험이나 불편감이 생기는 경우, 또는 연구와 관련된 손상이 발생할 경우, 위의 연구자에게 연락하여 주시기 바랍니다.
※연구에 참여하는 피험자로서의 권리에 관한 문의 및 상담 : UNIST 생명윤리위원회 행정간사 (Tel. 052-217-5214)</td></tr>
</table>

* 각각의 항목의 예시를 삭제하고 연구자 측에서 내용을 채워넣어야 합니다.

1. 연구의 배경 및 목적

본 연구는 대학생들의 발표불안(Public Speaking Anxiety, PSA)에 대한 가상현실(VR) 기반 면접 노출치료 효과를 검증하기 위한 것입니다. 발표불안은 학업 성취와 진로 준비에 부정적인 영향을 미치며, 특히 사회적 평가 압력이 큰 한국 대학생 집단에서 두드러집니다. VR 노출치료는 불안을 완화할 잠재력이 있으나, 지금까지 연구는 청중 수나 공간 규모와 같은 물리적 요인에 집중되어 사회적 상호작용의 역할은 충분히 검증되지 못했습니다. 따라서 본 연구에서는 질문 난이도와 면접관 반응을 교차 조합한 VR 환경을 통해 발표불안 완화 효과를 다차원적으로 분석하고, 사회적 상호작용이 VR 치료에서 어떤 기전을 통해 작용하는지를 규명하고자 합니다.

2. 예상 참여기간 및 본 연구에 참여하는 대략의 전체 피험자 수

본 연구에 예상되는 참여기간은 위원회 승인일로부터 2026 년 6 월 30 일 까지이며, 약 일주일 동안 20 분 가량의 설문과 30 분 가량의 VR 세션을 각 3 번 진행하고 이후 6 주 및 12 주 후 추적 조사를 받게 됩니다. 본 연구에 참여하는 피험자 수는 30%의 탈락율을 고려하여 총 92 명입니다.

3. 피험자 선정기준 및 제외기준

본 연구의 피험자로서의 선정기준과 제외기준은 아래와 같습니다.
[선정기준]
- 만 18 세 이상 대학생 또는 대학원생
- Personal Report of Public Speaking Anxiety-18 (PRPSA-18) 58 점 이상
- 한국어로 의사소통이 가능한 자
- 서면 동의서에 서명한 자

[제외기준]
- 심각한 공황장애, 사회불안장애, 주요우울장애 진단을 받은 자
- 명확한 자살사고가 확인된 자(PHQ-9 마지막 문항 1 점 이상)
- VR 기기 사용이 불가능한 신체적 제약이 있는 자 (심한 시력 장애, 전정기관 질환 등)
- 현재 발표불안에 대한 다른 심리치료나 약물치료를 받고 있는 자

4. 본 연구 참여에서 종료 시까지 받게 되는 검사 및 절차

- 연구 참여자는 스크리닝 및 기저선 평가를 받게 됩니다. 이 과정에서 발표불안 관련 설문지(PRPSA-18, PSAS, LSAS-SR, FNE-B)와 인구통계 정보를 작성하며, VR 사용 경험과 발표·면접 경험을 확인합니다. 또한 심박수와 피부전도(EDA) 같은 생체신호를 안정 상태에서 측정하고, 상반신 비디오 촬영과 음성 녹음을 통해 발표 시 행동 및 음성 패턴을 기초자료로 수집합니다.
- 앞으로 총 3 회의 세션에 참여하게 됩니다. 각 세션은 약 30 분 정도 소요되며, 준비와 마무리 시간을 포함합니다. 세션 동안 가상 환경에서 면접 상황이 재현되며, 다양한 형태의 질문과 반응이 주어질 수 있습니다.
- 세션 중에는 심박수와 피부전도 같은 생체신호가 연속적으로 측정되며, 상반신 촬영 영상과 음성이 함께 기록됩니다.
- 연구 참여 후 6 주와 12 주 시점에 추적 평가가 진행됩니다. 이때 다시 설문지를 작성하고, 실제 발표 경험과 연구 참여 경험에 대한 응답을 하게 됩니다.

5. 본 연구를 위해 피험자가 준수해야 하는 사항

본 연구 기간 동안 귀하가 준수해야 하는 사항은 다음과 같습니다.

- 연구진이 제시하는 일정에 맞추어 세션에 성실히 참석해 주셔야 합니다.
- VR 기기 사용으로 인한 어지럼증 등 방지를 위해 세션 전날 과도한 음주를 피하고 충분한 수면을 취해야 합니다.
- 연구 참여 중 불편한 점이 있을 경우 언제든지 연구자에게 문의하거나 참여를 중단할 수 있습니다.

6. 본 인간대상연구의 검증되지 않은 실험적인 측면

VR 기반 발표불안 치료는 일부 연구에서 효과가 보고되었으나, 본 연구에서 다루는 면접관의 사회적 반응과 같은 상호작용 요소가 발표불안 완화에 미치는 차별적 효과는 아직 과학적으로 충분히 검증되지 않았습니다.

7. 본 연구에 참여함으로써 피험자(피험자가 임부일 경우 태아, 수유부일경우 영유아)에게 미칠 것으로 예견되는 위험(부작용)이나 불편사항

참여 과정에서 VR 멀미, 눈의 피로·건조, 일시적 어지러움, 방향감각 혼란 등이 발생할 수 있습니다. 또한 발표 상황에서 불안이 일시적으로 증가하거나 회피 반응이 강화될 수 있습니다. 헤드셋과 센서 착용으로 인한 물리적 불편도 있을 수 있습니다.

8. 본 연구에 참여함으로써 기대되는 이익(이익이 없을 경우 그에 대한 명시)

직접적인 의학적 이익은 보장되지 않지만, 연구 참여자는 실제 평가 부담 없이 면접 상황을 체험하고 대처 기술을 훈련할 수 있는 기회를 얻게 됩니다. 이는 향후 발표나 면접 상황에서 불안 완화에 도움을 줄 수 있습니다.

9. 본 연구에 관련된 손상이 발생하였을 경우 피험자에게 주어질 보상이나 치료 방법

연구 참여 중 손상이 발생할 경우 연구책임자에게 즉시 보고되며, 연구진은 적절한 조치를 취하고 필요한 경우 상담이나 치료로 연계합니다. 심각한 이상반응은 24 시간 이내에 연구책임자와 IRB 에 보고됩니다. 보상은 IRB 규정에 따릅니다.

10. 본 연구에 참여함으로써 받게 되는 금전적 보상 유무, 참여 정도에 따른 조정 정도 및 피험자에게 추가적으로 발생이 예상되는 비용

본 연구에 참여함으로써 받게 되는 금전적 보상으로는 3 회의 방문 및 추가적인 설문 응답에 대해 교통비 및 소정의 참여비 50,000 원이 지급되며 피험자에게 추가로 발생하는 별도의 비용은 없습니다.

11. 연구 참여의 제한

다음에 해당되는 경우, 귀하는 귀하의 동의 없이도 본 연구의 참여로부터 제한될 수 있습니다.
A. 연구자의 지시를 따르지 않고 중대한 프로토콜 위반을 반복할 경우
B. 연구 참여와 관계 없는 중대한 질환이 발생할 경우
C. 심한 공황 발작, VR 멀미로 인한 지속적 구토 등 심각한 이상반응이 발생할 경우

12. 피험자가 연구 참여에 동의하지 않더라도 불이익이나 차별을 받지 않는다는 것 / 피험자가 자발적으로 연구 참여를 동의한 경우라도 자유의사에 의해 언제든지 이를 철회할 수 있다는 내용(단, 이와 같은 철회가 연구참여 개시 이전에 이루어지고 시험참가에 대한 보상이 있었던 경우 보상금의 반환 여부)

본 연구에 참여 여부를 결정하는 것은 귀하에게 달려있습니다. 귀하는 연구 참여를 결정하였다 하더라도, 언제든지 동의철회를 통해 연구 참여를 그만둘 수 있습니다. 귀하가 본 연구에 참여하지 않거나 참여 중단을 하더라도 아무런 불이익이나 차별을 받지 않습니다. 다만, 연구를 중도에 철회하는 경우에는 피험자비를 지급받을 수 없습니다. 만약, 귀하가 연구 참여에 대한 동의를 철회하고자 한다면, 연구책임자는 연구와 동의철회의 이유에 대해 묻겠지만 귀하가 그에 동의하지 않는다면 어떠한 이유도 묻지 않을 것입니다. 또한 동의철회 시점에서의 연구결과 데이터를 수집하는 것에 대해 귀하에게 동의를 구할 것이나 이 역시 귀하가 동의하지 않으면 귀하에 대한 어떠한 새로운 자료도 요구하지 않을 것입니다.

13. 개인정보 수집과 보호대책(이용 . 관리 . 파기)

본 연구에서는 성별, 연령, 연락처, 생체신호, 영상 및 음성 데이터를 수집하고자 합니다. 연구에 필요한 최소한의 정보만 수집되며 연구에 필요하지 않은 개인정보는 수집하지 않을 것입니다. 수집된 개인정보는 고유 코드화·암호화되어 관리되며 연구 목적 외에는 사용되지 않습니다. 연구 종료 후 규정에 따라 안전하게 폐기되거나 추가 연구 활용 시 별도 동의를 받습니다. 귀하는 언제든 본인의 개인정보에 대해 열람. 정정. 삭제를 요구할 수 있는 권리를 가지고 있습니다.

14. 신분의 비밀보장(자료의 열람 권한 설정 및 보관, 관리, 폐기 및 연구 결과 발표 시 피험자의 신원 보호)

귀하의 신원을 파악할 수 있는 기록은 비밀유지가 되며 공개적으로 타인에게 열람되지는 않습니다. 다만, 관련법이나 규정에 의해 허용되는 범위 안에서 연구 실시 절차와 자료의 신뢰성을 검증하기 위해 심사위원회 및 정부기관에 의해 귀하의 자료를 직접 열람할 수 있지만 이 경우에도 최대한 비밀유지가 되도록 할 것입니다. 본 연구의 결과가 출판될 경우 귀하의 신원을 비밀 상태로 유지될 것입니다.

15. 본 연구의 지속 참여 의지에 영향을 줄 수 있는 새로운 정보가 얻어지면 적시에 피험자 본인 또는 대리인에게 알려드릴 것입니다.

16. 연구에 대한 추가적인 정보를 얻고자 하거나 연구와 관련 있는 손상이 발생한 경우 접촉해야 하는 사람과 연락처

본 연구의 참여와 관련하여 문의 및 불만 사항이 있으신 경우에는 언제든지 연구자에게 연락하여 주시기 바랍니다.
< 연구자 성명: 김명성, 연락처 (☎) 010-9942-5148 >

연구대상자 동의서

**연구과제명 :**	사회평가적 위험 상황에서의 가상현실 면접 노출치료가 발표불안에 미치는 효과: 상황적 요소와 상호작용 요소를 고려한 무작위 대조 시험
**IRB 승인번호 :**	피험자에게 제공 시 IRB 승인번호를 기입하여 사용

본 동의서에 기술 된 목적 이외의 다른 연구에 본인의 연구데이터(샘플) 또는 검체를 2 차적으로 사용하는 것에 동의하십니까?

	**추가적인 동의 없이, 데이터(검체)의 개인식별정보를 포함한 상태로 이용**
	**추가적인 동의 없이, 데이터(검체)의 개인식별정보를 코드화하여 피험자 식별이 추적 가능한 상태로 이용**
	**추가적인 동의 없이, 데이터(검체)의 개인식별정보를 완전히 제거한 상태로 이용**
	**다른 종류의 연구에 사용할 경우, 본인의 추가 사전 동의 후 진행 바랍니다.**
	**다른 목적으로 사용하는 것에 동의하지 않습니다.**

본인은 본인과 연구자 및 UNIST 사이에 본인의 연구 참여 결정에 영향을 줄 수 있는 어떠한 관계도 없습니다.

본인은 연구 관련자로부터 이 동의서에 대한 설명을 들은 후, 본인이 직접 읽고 이해하였으며 모든 질문에 대한 답변을 들었습니다. 또한 아무런 강압 없이 자발적으로 동의서를 작성하며 이에 본 연구에 참여한다는 것을 서명으로 확인합니다.

(날짜 및 서명은 반드시 자필로 작성)

**피험자**	(성명)__________	(자필서명)__________	(서명일)__________
**법정 대리인(필요 시)**	(성명)__________	(자필서명)__________	(서명일)__________
	(피험자와의 관계) ______________		
**입회인(필요 시)**	(성명)__________	(자필서명)__________	(서명일)__________
**연구책임자**	(성명)__________	(자필서명)__________	(서명일)__________

본 연구는 UNIST IRB 에서 심의하여 승인한 동의서만을 이용합니다.
