# Supplementary material for: Virtual reality exposure therapy with graded interviewer reactions for public speaking anxiety in university students: a randomized controlled trial protocol
Source: Trials. 2026 May 19;27:485. doi: 10.1186/s13063-026-09779-0 (PMC13352607; doi:10.1186/s13063-026-09779-0)
Supplement: Supplementary file 1 — Supplementary Material 1. [file 13063_2026_9779_MOESM1_ESM.zip › Supplementary4_IRB Documentation (English Translation Cover Letter).docx]

INSTITUTIONAL REVIEW BOARD APPROVAL DOCUMENTATION

*The study title in this manuscript differs from the IRB-approved title to better align with the journal's scope and enhance reader comprehension. The IRB-approved title broadly describes the research context, while the manuscript title specifically emphasizes the key intervention component and target population for clearer communication in the scientific literature.

IRB-Approved Study Title (Korean): 사회평가적 위험 상황에서의 가상현실 면접 노출치료가 발표불안에 미치는 효과: 상황적 요소와 상호작용 요소를 고려한 무작위 대조 시험

IRB-Approved Study Title (English): Effects of Virtual Reality Interview Exposure Therapy on Public Speaking Anxiety in Social-Evaluative Threat Situations: A Randomized Controlled Trial Considering Situational and Interactive Factors

Manuscript Title (for journal submission): Virtual Reality Exposure Therapy with Graded Interviewer Reactions for Public Speaking Anxiety in University Students: A Randomized Controlled Trial Protocol

IRB Approval Number: UNISTIRB-25-049-A

Project Number: 2025-08-005 (Initial), 2025-10-007 (Amendment)

Principal Investigator: Dooyoung Jung, Professor

Affiliation: UNIST Healthcare Center, Ulsan National Institute of Science and Technology (UNIST)

Review Type:

- Initial Review: Approved on October 16, 2025

- Amendment Review: Approved on October 31, 2025

Approval Period: October 17, 2025 – December 31, 2026

Review Board: UNIST Institutional Review Board (IRB)

IRB Chair: Byung Heon Kang

Study Type: Human subjects research

Key Approval Details:

- Research protocol Version 1.0 (initial), Version 1.1 (amended)

- Informed consent form approved

- Participant recruitment materials approved

- Study questionnaires and interview tools approved

The attached documents include:

1. Initial IRB approval notification (dated October 16, 2025)

2. Amendment IRB approval notification (dated October 31, 2025)

This study complies with the Korean Bioethics and Safety Act and adheres to the Declaration of Helsinki principles for research involving human subjects.
