# Supplementary material for: Virtual reality exposure therapy with graded interviewer reactions for public speaking anxiety in university students: a randomized controlled trial protocol
Source: Trials. 2026 May 19;27:485. doi: 10.1186/s13063-026-09779-0 (PMC13352607; doi:10.1186/s13063-026-09779-0)
Supplement: Supplementary file 1 — Supplementary Material 1. [file 13063_2026_9779_MOESM1_ESM.zip › Supplementary7_Funding Documentation(English Translation Cover Letter).docx]

RESEARCH FUNDING DOCUMENTATION

IMPORTANT NOTE ON PROJECT SCOPE: This manuscript represents a focused sub-study within a larger funded project. The parent project encompasses comprehensive digital therapy content development for various anxiety symptoms, while this protocol paper specifically addresses one component: virtual reality exposure therapy for public speaking anxiety in university settings.

RELATIONSHIP BETWEEN PARENT PROJECT AND CURRENT MANUSCRIPT:

Parent Project (Funded):

Title: "Development and Verification of Digital Therapy Contents for Anxiety Control through Psychiatry-Art Convergence Research"

Scope: Comprehensive digital therapy interventions for multiple anxiety subtypes:

- Generalized anxiety → Meditation-based interventions

- Panic disorder/agoraphobia → Place-specific exposure therapy

- Social anxiety disorder → Public speaking and job interview exposure therapy

Current Manuscript (Sub-study):

Title: "Virtual Reality Exposure Therapy with Graded Interviewer Reactions for Public Speaking Anxiety in University Students: A Randomized Controlled Trial Protocol"

Scope: In-depth investigation of one specific component—interview/presentation exposure therapy for public speaking anxiety in university student population

Rationale for Focused Protocol:

This protocol paper reports a rigorous randomized controlled trial examining the specific contribution of graded interviewer reactions (positive → neutral → negative) in VR-based exposure therapy. This represents a theoretically and methodologically distinct investigation within the broader funded project, warranting separate protocol publication to:

1. Establish detailed methodology for this specific intervention component

2. Enable prospective trial registration and transparent reporting

3. Facilitate replication and future meta-analyses

4. Address a focused research question suitable for a single RCT

The findings from this sub-study will inform the development and refinement of the social anxiety component within the larger digital therapy platform.

FUNDING AGREEMENT SUMMARY

Research Program: Science and Technology-Humanities Convergence Research Program

Funding Agency: National Research Foundation of Korea (NRF)

Ministry: Ministry of Science and ICT (MSIT), Republic of Korea

Main Project Title (Korean): 정신의학-예술 융합연구를 통한 불안증상 조절 디지털치료 콘텐츠 개발 및 실증

Main Project Title (English): Development and Verification of Digital Therapy Contents for Anxiety Control through Psychiatry-Art Convergence Research

Lead Institution: Korea University (main project coordinator)

Collaborating Institutions:

- Ulsan National Institute of Science and Technology (UNIST) - Co-Principal Investigator: Hwang Kim

- Korea National University of Arts

- Medimind Co., Ltd.

UNIST Sub-project Funding:

- Principal Investigator: Hwang Kim, Associate Professor, UNIST

- Total Project Period: June 1, 2022 – December 31, 2026 (54 months)

- Multi-year Agreement Period: June 1, 2022 – December 31, 2024

- Current Year Research Period: January 1, 2024 – December 31, 2024

Research Funding Breakdown (Unit: 1,000 KRW):

Year 1 (2022): 75,000 KRW

Year 2 (2023): 84,000 KRW

Year 3 (2024): 47,600 KRW

Year 4 (2025): 110,000 KRW

Year 5 (2026): 100,000 KRW

Total: 416,600 KRW (approximately $312,450 USD)

Agreement Date: April 2024

Contract Authority: National Research Foundation of Korea (NRF), acting on behalf of the Minister of Science and ICT

Agreement Parties:

- Funding Agency: National Research Foundation of Korea (President)

- Research Institution: Ulsan National Institute of Science and Technology (President)

- Principal Investigator: Hwang Kim, Associate Professor

This funding agreement is governed by the Korean National R&D Innovation Act and its implementing regulations. The attached documents include:

1. Standard Research Agreement between NRF and UNIST

2. Annual Research Progress Report and Plan (STEAM Program, 2024)

The current protocol paper represents a sub-study within this larger funded project, specifically focusing on virtual reality exposure therapy for public speaking anxiety among university students.
