# Supplementary material for: Virtual reality exposure therapy with graded interviewer reactions for public speaking anxiety in university students: a randomized controlled trial protocol
Source: Trials. 2026 May 19;27:485. doi: 10.1186/s13063-026-09779-0 (PMC13352607; doi:10.1186/s13063-026-09779-0)
Supplement: Supplementary file 1 — Supplementary Material 1. [file 13063_2026_9779_MOESM1_ESM.zip › Supplementary9_Participant Information and Consent Forn (English Translation_Cover_Letter).docx]

PARTICIPANT INFORMATION SHEET AND INFORMED CONSENT FORM

This supplementary document provides an English-language overview of the Participant Information Sheet and Informed Consent Form that were originally approved by the UNIST Institutional Review Board (IRB). The purpose of this document is to enhance transparency for non-Korean reviewers by clarifying the structure, scope, and ethical components of the participant materials included in the study protocol.

**Purpose of This Supplementary Document**

The materials in this document summarize the content of the officially approved Korean Participant Information Sheet and Informed Consent Form. The original documents, which are provided in full, are the legally binding versions presented to participants. This English summary enables external reviewers to clearly understand the ethical safeguards, procedures, and regulatory compliance relevant to the study. The summary aligns with national human subjects research regulations and international reporting standards to support ethical review, reproducibility, and protocol assessment.

**Summary of Key Elements Contained in the Participant Information Sheet**

The participant information materials include the following components:

1. Background, rationale, and objectives of the study
2. Expected participation timeline and total sample size
3. Inclusion and exclusion criteria
4. Study procedures and assessments
5. Participant responsibilities
6. Potential risks and discomforts
7. Anticipated benefits
8. Compensation and reimbursements
9. Procedures for reporting adverse events and available medical support
10. Data protection, confidentiality, and storage measures
11. Voluntary participation and the right to withdraw without penalty
12. Contact information for the research team and the IRB

**Summary of the Informed Consent Form**

- The Informed Consent Form includes the following elements:
- Confirmation that participants received, read, and understood both written and verbal explanations.
- Options regarding secondary use of data, including distinctions among anonymized, coded, and identifiable data formats.
- Signature sections for the participant, legal representative if applicable, investigator, and witness.
- Statement that only IRB-approved consent materials will be used.

**Included Files**

This submission includes the following original Korean documents, each carrying IRB approval:

1. Participant Information Sheet (Korean original)
2. Participant Informed Consent Form (Korean original)

These documents are provided to ensure ethical transparency, facilitate review by non-Korean evaluators, and support methodological reproducibility.
